# Comparative metagenomics indicates metabolic niche differentiation of benthic and planktonic *Woeseiaceae*

**DOI:** 10.1186/s40793-025-00732-3

**Published:** 2025-06-17

**Authors:** Tomeu Viver, Katrin Knittel, Rudolf Amann, Luis H. Orellana

**Affiliations:** 1 Department of Molecular Ecology, Max Planck Institute for Marine Microbiology, Bremen, Germany; 2 Ecological Genomics Group, Max Planck Institute for Marine Microbiology, Bremen, Germany

**Keywords:** Metagenomics, Sediment microbial communities, Helgoland, Polysaccharide utilization loci, Chemolithoautotrophy, Long-read sequencing

## Abstract

**Background:**

Benthic microbiomes exhibit remarkable temporal stability, contrasting with the dynamic, substrate-driven successions of bacterioplankton. Nonetheless, understanding their role in carbon cycling and interactions between these two microbial communities is limited due to the complexity of benthic microbiomes.

**Results:**

Here, we used a long-reads (LRs) metagenomic approach to examine benthic microbiomes and compared them to the microbiomes in the overlaying water column and on particles, sampled at the same site and time off the island Heligoland in the North Sea. Although the diversity is vast in marine sediments, we recovered high quality metagenome assembled genomes (MAGs). Based on taxonomy and metabolic annotation of predicted proteins, benthic microbiomes are distinctly different from pelagic microbiomes. When comparing the 270 MAGs from free living and particle attached microbes from the water column to 115 MAGs from sediments only 2 MAGs affiliated to *Acidimicrobiia* and *Desulfocapsaceae* were shared at species level. Although, we recovered MAGs with the same taxonomic annotation in pelagic and benthic microbiomes, their metabolic potentials were different. A prominent example was the family *Woeseiaceae* that was among the most abundant taxa in the sediments. In benthic *Woeseiaceae* MAGs, we found polysaccharide utilization loci (PULs), predicted to target laminarin, alginate, and α-glucan. In contrast, pelagic *Woeseiaceae* MAGs were only recovered in the particle attached but not in the free-living fraction, and lacked PULs. They encoded a significantly more sulfatases and peptidases genes. Additionally, while genes involved in iron acquisition, gene regulation, and iron storage were widespread in *Woeseiaceae* MAGs, genes linked to dissimilatory iron reduction were mostly restricted to benthic *Woeseiaceae*, suggesting niche-specific adaptations to sediment redox conditions. Both, benthic and pelagic particle-attached *Woeseiaceae* MAGs encoded pilus TadA genes, which are essential for adhesion, colonization, and biofilm formation.

**Conclusions:**

LR sequencing is currently the most valuable tool for analyzing highly diverse benthic microbiomes. The small overlap of MAGs from water column and sediments indicated a limited bentho-pelagic coupling. The data suggest that *Woeseiaceae* have habitat-specific metabolic specialization: while benthic *Woeseiaceae* possess the metabolic capabilities to utilize fresh organic compounds like laminarin derived from algae blooms, and to perform dissimilatory nitrate, nitrite and iron reduction for gain energy, particle attached *Woeseiaceae* from the water column may be specialized in degrading protein-rich and sulfated organic matter likely reflecting adaptation to the different types of organic matter and redox conditions in sediments vs. the water column.

**Supplementary Information:**

The online version contains supplementary material available at 10.1186/s40793-025-00732-3.

## Introduction

Gaining insight into the ecological niches that allow the colonization, proliferation, and establishment of microbial communities in a given environment remains a fundamental challenge in microbial ecology [1]. A comprehensive understanding of the environmental conditions (abiotic factors), the biotic interactions among organisms, and the diverse microbial metabolic pathways are imperative to delineate microbial ecological niches. The niche theory has mainly gained significance in elucidating the adaptability of microorganisms within marine environments, specifically concerning their responses to seasonal variations [2], that for the most part, are driven by environmental factors, such as temperature, salinity [3, 4] and substrate availability [5–7]. Nonetheless, little is known about the metabolic differentiation of taxonomically related microorganisms living in marine sediments vs. in pelagic environments. The majority of studies addressed this question had been limited to 16 S rRNA gene amplicon sequencing [8, 9].

While bacterioplankton exhibited considerable variability and dynamics in microbial populations, sediment-dwelling microbial communities demonstrate remarkable temporal stability [8, 10, 11]. For instance, in the North Sea, phytoplankton spring blooms have been shown to cause substrate-based successions of pelagic taxa such as *Polaribacter*,* Roseobacter*, *Flavobacteria*, *Verrucomicrobia*, SAR92 and archaeal populations [2, 6, 12–15]. In contrast, sediment communities exhibit a much higher stability even in the presence of strongly seasonal phytodetrital inputs, featuring dominant taxa like *Woeseiaceae*, *Desulfobacterota*, and *Planctomycetota* [8, 11].

Members of the *Woeseiaceae* family have been recognized as a significant component of the benthic microbial communities dominating in various settings, ranging from shallow-sea coastal sediments [16–21] to deep ocean seafloor [22–24], hydrothermal vent chimneys [25], marine solar salterns [26], as well as in both, oxic and anoxic environments [27]. Ecological and phylogenetic studies have described the *Woeseiaceae* family as a widely distributed group within the class *Gammaproteobacteria*, exhibiting significant prevalence in marine sediments worldwide [23]. Recently, Moncada and colleagues [21] showed that *Woeseiaceae* cells preferentially grow firmly attached to grains where they are most active compared to those living in the porewater or being loosely attached. Genome-based metabolic predictions, metagenomic and metatranscriptomics analyses indicated that species within the *Woeseiales* are habitat generalists [28] being metabolically versatile, encompassing facultative sulfur- and H_2_-based chemolithoautotrophy to obligate chemoorganotrophy [16, 23, 26, 29, 30]. The oxidation of sulfur and other reduced sulfur intermediates such as thiosulfate (S₂O₃²⁻) is mediated via the Sox pathway and likely linked to carbon fixation via the Calvin-Benson cycle [30]. Their chemoorganotrophic metabolism involved the utilization of mono- and polysaccharides along with proteolytic activity, suggesting a significant role in the degradation of protein-rich and sulfated organic matter, including remnants of cell membranes or cell walls [16, 23, 29]. Their metabolic versatility is particularly evident in their ability to switch between electron acceptors during oxic-anoxic transitions. In addition to the use of oxygen, *Woeseiaceae* species possess a truncated denitrification pathway and contribute to the release of N_2_O from sediments [23, 31]. These diverse metabolic pathways suggest substantial ecological diversification among *Woeseiaceae* species. Yet, due to limited cultivability and the lack of high-quality genome information, the genetic basis of their niche differentiation and its implications for organic matter recycling remain insufficiently understood.

Here, we applied a deep long-read (LR) metagenomic approach to explore the diversity and metabolic capabilities of the sediment microbial communities in the upper layers of oxic coastal sandy sediment samples from Helgoland on five sampling dates in 2018/2019. LR metagenomics offers the advantage of recovering full-length 16 S rRNA gene sequences, allowing accurate taxonomic analyses of the complete sediment microbial community [32]. Moreover, we compared sediment microbial diversity with those from the overlaying water column during the phytoplankton spring bloom, including free-living and particle-attached bacteria. The recovery of high-quality *Woeseiaceae* MAGs from sediments and overlaying water column samples allowed us to detect niche-specific metabolic capabilities at the species level.

## Materials and methods

### Study site and sampling

Samples were obtained by scientific divers from the Alfred Wegener Institute (Bremerhaven, Germany) using push cores at a shallow coastal site at Helgoland Roads, North Sea (German Bight; 54.182°N, 7.902°E) in 2018 and 2019. Water depths ranged over the tidal cycle between ∼ 6 and ∼ 8 m. The sediments are characterized by fine and medium sand with variable portions of coarse to very coarse sand (for details see [8, 11, 18]). We selected five surface (0–2 cm depth) sediment samples collected on March 7th 2018 (referring as sample Mar18), April 17th 2018 (Apr18), May 15^t^h 2018 (May18), September 8th 2018 (Sep18) and January 18th 2019 (Jan19) for metagenomics analysis (Table 1). Surface seawater temperature ranged between 3.4 °C in winter to 19 °C in September. Salinity was measured between 33 and 34 PSU. The total organic carbon content was low with 0.07 wt % to 0.7 wt %. The highest chlorophyll *a* concentration was measured in September with 2.5 µg ml^− 1^ sediment (for details see Table S1). Furthermore, samples retrieved from the same site on March 17th, April 20th, and May 24th in 2016 as well as from site NoaH-B (53.987°N, 6.868°E), located about 80 km apart from Helgoland, North of the East Frisian Islands, were used for comparison. NoaH-B was sampled with a Van Veen grab during a cruise with RV Heincke (He417) at 28.6 m water depth [18].

### Metagenome analyses using PacBio long-reads

DNA was extracted from sediment samples (0–2 cm depth) according to Zhou et al. [33] with minor modifications [8, 11]. The extracted DNA was quantified using fluorometry (Quantus, Promega), and the quality was assessed using capillary electrophoresis (Agilent FEMTOpulse). Metagenomic libraries were prepared using the HiFi SMRTbell^®^ Libraries from Ultra-Low DNA Input protocol. HiFi SMRT sequencing was done for 30 h on a Sequel IIe device at the Max Planck Genome Center in Cologne, Germany. DNA quantification, library preparation, and sequencing were performed following the manufacturers’ protocols. Ultra-low adapter trimming was performed using PacBio SMRT Link version 25.1.0, using default settings. Metagenomic LR quality was assessed using Nanoplot v1.39.0 [32]. Beta-diversity analysis was conducted using MASH distance (Mash v1.1 [35]), using default parameters. MASH distance values were visualized in an NMDS plot using the vegan library [36], and a PERMANOVA test using adonis2 method was applied to assess the statistical significance between sediment and water column samples. Taxonomic classification of metagenomic LRs was performed using Kaiju v1.6.3 [37] and Metabuli v1.0.5 [38], both executed with default parameters. Kaiju was used to identify eukaryotic metagenomic reads, while taxonomic classification of prokaryotic reads was performed using Metabuli.

16 S rRNA genes were extracted from metagenomic LRs using barrnap v0.9 (https://github.com/tseemann/barrnap) and clustered in operational taxonomic units (OTUs) at 98.7% similarity (minimum threshold discriminating species suggested by Stackebrandt and Goebel (1994) [39]) using uclust v1.2.22 [40, 41]. Singleton and doubleton OTUs were removed from the analysis. Representative OTU sequences were imported into the non-redundant SILVA REF 138.1 database [42]. Sequences were aligned using the SINA v1.3.1 aligner [43] implemented in the ARB program package v6.0.6 [44]. Rarefaction analyses and alpha diversity indices were conducted using PAST v4.03 [45].

### Metagenomic assembly and binning

Metagenomic assembly and binning were performed separately for each PacBio LR metagenome and as on co-assembled datasets to assess how sequencing effort influences the number of MAGs recovered from sediment samples. To this end, we employed two co-assembly strategies based on subsampling of the metagenomic dataset generated in this study (*n* = 9). In the first strategy, metagenomic files were concatenated in chronological order, starting with the five metagenome replicates from the Mar18 sample, followed by samples from Apr19 to Jan19. In the second strategy, all samples were first concatenated into a single dataset, from which reads were randomly subsampled to match the total data size (in Gbp) of the first strategy. Single and concatenated metagenomes were assembled with Flye v2.8.1 [46] using the options *pacbio-hifi* and *meta*. For each metagenomic dataset, the sequencing depth was calculated mapping long-reads using minimap2 v2.17 [47] with the *map-hifi* option and using the *jgi_summarize_bam_contig_depths* script from MetaBAT v2.2.15 [48]. Contigs larger than 2,000 bps were binned using MaxBin v2.2.7 [49] and MetaBAT v2.2.15 [48]. MAG completeness and contamination values were calculated using CheckM v1.1.6 [50] and CheckM2 v0.1.3 [51]. The quality for each MAG was estimated as the composite index ‘completeness– 5 x contamination’ of the CheckM v1.1.6, following established standards for genome quality assessment [13, 14, 31]. MAGs with a quality score ≥ 50% based on this metric were selected for further analysis. MAGs obtained from the co-assembly of all samples generated in this study (*n* = 9) were used for comparative analyses. dRep v3.2.2 was used to de-replicate MAGs in genomospecies (gspp) with the options -pa 0.96 (ANI threshold to form primary clusters) [52]. Gene prediction from LR metagenomes was conducted using Prodigal v2.6.3 [53], and representative gspp MAGs were selected for relative abundance estimation.

***MAGs relative abundance estimation using PacBio long reads.***

To measure the average number of universally conserved single-copy genes in the LRs metagenomes (i.e., genome equivalents), we used the script *HMM.essential.rb* (with the “metagenome” option) from enveomics collection [54]. Genome equivalents were calculated for each metagenomic dataset as the average number of genes predicted for each set of universally conserved single-copy genes. The sequencing depth for each MAG was estimated mapping the unassembled LRs using minimap2 v2.17 [47] with the *map-hifi* option, and filtering mapped LRs at ≥ 95% identity using the *jgi_summarize_bam_contig_depths* script from MetaBAT v2.2.15 [48]. These average sequencing depth values were normalized using the genome equivalents determined for each metagenome [32].

***Metagenomes and MAGs recovery from water column metagenomes.***

Microbial diversity and metabolic profiles of the water column samples collected during the 2018 Helgoland spring bloom were described in Wang et al., 2024 [55], from which the metagenomes and MAGs used for comparison in this study were obtained. The sampling dates were either identical to or within a few days of the sediment sampling. This slight discrepancy in timing can be considered negligible, given the high stability of the benthic microbial community composition observed throughout the one-year sampling period. All MAGs were dereplicated using dRep v3.2.2 [52] with a 96% ANI cut-off. To estimate MAG relative abundances, metagenomic reads were mapped against MAGs using the BLASTn option of the aligner Magic-BLAST + v2.12.0 with default settings [56]. Reads matching with higher than the 95% sequence identity and an (alignment length) / (query read length) greater than 0.9 were used to determine the sequencing depth for all representative MAGs in each metagenomic dataset. The resulting sequencing depth values were truncated to the middle 80% (TAD80) (i.e., the upper and lower 10% of outliers were removed) using a Python script from https://github.com/rotheconrad/00_in-situ_GeneCoverage [14, 57]. Finally, to estimate the relative abundance with respect to the abundance of bacterial and archaeal communities, each TAD80 value was normalized by the “genome equivalents” value estimated using MicrobeCensus v1.1.0 [30, 58].

### MAGs phylogenetic classification

Phylogenetic analyses of MAGs were performed using the GTDB-tk v2.1.1 (release 207_v2) with the *classify_wf* pipeline [59]. The alignment of housekeeping proteins produced in GTDB-tk was used for phylogenetic tree reconstruction using IQ-TREE v1.6.12 [60]. Visualization and editing of the phylogenetic trees were conducted using iTol [61].

All 16 S rRNA genes were extracted from LR MAGs using barrnap v0.9 (https://github.com/tseemann/barrnap). The sequences were imported into the latest updated Living Tree Project database (LTP_06_2022) containing all sequences of the type strains classified reported until June 2022 [62] and in the SILVA REF 138.1 database [42]. Sequences were aligned using SINA v1.3.1 [43] in ARB v6.0.6 [44], and manually checked to improve the alignment and to finally perform the phylogenetic analyses using ARB package v6.0.6 [44].

The ANI and fraction of genome shared between all MAGs was calculated using *ani.rb* [54] based on the BLASTn [63].

### LR metagenomes and MAGs gene annotation and PUL prediction

Annotations of CAZymes of protein-coding genes predicted from LR metagenomes and MAGs were annotated using the dbCAN v10 database [64] and DIAMOND searches [65] against the CAZy database v04242021 (E-value ≤ 1e-20) [66]. Only genes positive against both dbCAN and the CAZY database were considered reliably annotated as CAZymes. SulfAtlas v2.3.1 [67] was used to annotate sulfatases using DIAMOND (E-value ≤1e-20). The MEROPS v12.4 database [68] was used to annotate peptidases using DIAMOND (E-value ≤1e-20). Moreover, predicted genes from MAGs were annotated using the SwissProt and TrEMBL databases (downloaded in September 2022 [69]) and DIAMOND v0.9.31 with default settings [65]. Also, proteins were annotated using the Kyoto Encyclopedia of Genes and Genomes (KEGG) database [70] and DRAM v1.0 (Distilled and Refined Annotation of Metabolism) [71]. TonB-dependent transporters (TBDTs) were predicted by HMMscan against TIGRFAM profiles TIGR01352, TIGR01776, TIGR01778, TIGR01779, TIGR01782, TIGR01783, TIGR01785, TIGR01786, TIGR02796, TIGR02797, TIGR02803, TIGR02804, TIGR02805, TIGR04056 and TIGR04057 (E-value ≤1E − 10). SusD genes were identified by HMMscan against the Pfam profiles PF12741, PF12771, PF14322, PF07980. Potential iron metabolisms were predicted using FeGenie v1.0 [72].

## Results

### Metagenome description and benthic microbial diversity

The long-read (LR) metagenomic approach covered the sampling period from March 7th, 2018 to January 18th, 2019, at five time points. For evaluating of the effect of sequencing depth, five replicates were sequenced from the initial sample (Mar18_A1 to A5). The average output for each metagenomic sample was 30.4 Gbp [interquartile range (IQR): 27–31 Gbp] and average read length of 5.1 kbp (IQR: 3.2–6.5 kbp) (Table 1 and S2). Thus, in this study a total of 274 Gb of LR PacBio HiFi sequencing data were generated. Beta diversity analysis based on k-mer distances revealed values ranging from 0.063 to 0.085 (on a scale from 0, indicating identical composition, to 1, indicating complete dissimilarity), underscoring the high similarity among sediment metagenomes (Fig. S1 and Table S3) [8]. The distance values among the Mar18 replicates were lower (average 0.067 ± 0.009) when compared with the other sampling points (average 0.08 ± 0.003). Based on the taxonomic affiliation of unassembled LRs, most of the sequenced metagenomic samples corresponded to bacterial sequences, and the proportion assigned to eukaryotes ranged between 6.3% and 6.9% (Table S2).

To obtain a broad overview of the community composition, we first exploited the recovery of full-length SSU rRNA gene sequences from all unassembled LRs. A total of 26,082 sequences (average 1,457 ± 143 bp length) were extracted from unassembled LRs, and after clustering the sequences at ≥ 98.7% identity, 1,732 OTUs were identified (Table 1). Of these, 1,618 were affiliated to *Bacteria*, 8 to *Archaea*, and 106 to *Eukarya* (Table S4). Despite the high sequencing effort, the non-saturated rarefaction curves of each metagenome individually indicated a low metagenomic community coverage of the bacterial populations (Fig. S2A). Nevertheless, rarefaction reached saturation by combining all metagenomes (Fig. S2B). The taxonomic classification of OTUs indicated that the most abundant classes were *Gammaproteobacteria* and *Alphaproteobacteria* (average relative abundance of 32 ± 2.6% and 18 ± 4.7%, respectively), followed by *Bacteroidia* (5.1 ± 1.3%), *Planctomycetes* (4.5 ± 0.5%), and *Acidimicrobiia* (4.3 ± 1.0%) (Fig. S3 and Table S5). At the OTU level, a new archaeal *Candidatus* Nitrosopumilus sp. was the most abundant species, comprising 3.8 ± 1.2% of the microbial community. Interestingly, within the class *Gammaproteobacteria*, the family *Woeseiaceae* comprised the most diverse family (represented by 50 OTUs) and reached a relative abundance of 7.6 ± 0.9%, similar to what was previously observed using 16 S rRNA sequence amplicons [8] (Table S6). In the replicated Mar18 samples, eukaryote OTUs accounted for 19.5 ± 2.5% of the total community, while in all other samples, less than 10.6% (Table 1 and S2). The most abundant Eukaryotes were assigned to copepods, the worm *Monostilifera*, and the animal parasite *Acetosporea*. At the class level, similar relative abundances for bacterial and archaeal groups were observed in all samples based on 16 S gene amplicons, full-length 16 S extracted from long-reads, and unassembled total metagenomic reads methodologies (Fig. S4). At the family and genus levels, the taxonomic distribution and relative abundance of bacterial and archaeal 16 S rRNA sequences in metagenomes were statistically similar to those previously observed using amplicon 16 S rRNA amplicon sequencing [8] (Fig. S5).

**Table 1:** Long-Read metagenomic statistics of Helgoland sediment samples

Sample	Mar18_A1	Mar18_A2	Mar18_A3	Mar18_A4	Mar18_A5	Apr18	May18	Sep18	Jan19
**Sampling day**	2018-03-07	2018-03-07	2018-03-07	2018-03-07	2018-03-07	2018-04-17	2018-05-15	2018-09-05	2019-01-18
**Metagenome size (Gbp)**	35	30	31	27	39	27	26	31	28
**No. reads**	3,153,305	3,706,560	3,298,344	3,434,288	3,065,523	2,569,567	2,692,975	3,390,715	3,115,280
**No. bps**	1.8 × 10^10^	1.5 × 10^10^	1.6 × 10^10^	1.4 × 10^10^	2.04 × 10^10^	1.43 × 10^10^	1.37 × 10^10^	1.64 × 10^10^	1.45 × 10^10^
**Average read length (bps)**	5,801	4,212	5,019	4,161	6,648	5,591	5,091	4,837	4,647
**No. 16 S rRNA sequences**	2,495	2,658	2,583	2,366	3,474	2,143	1,647	2,710	2,263
**No. OTUs**	831	835	819	815	976	783	635	802	716
**Dominance (D)**	0.007	0.009	0.008	0.009	0.008	0.006	0.006	0.007	0.008
**Shannon (H)**	5.93	5.87	5.87	5.84	6.02	6.03	5.88	5.92	5.81
**Archaea fraction (OTUs)**	2.78%	3.80%	3.48%	3.99%	4.60%	2.94%	1.90%	2.39%	3.46%
**Bacteria fraction (OTUs)**	78.76%	74.56%	76.12%	74.72%	79.87%	91.09%	87.47%	94.19%	93.33%
**Eukaryotic fraction (OTUs)**	18.46%	21.64%	20.40%	21.29%	15.54%	5.97%	10.63%	3.42%	3.21%

### Diversity and novelty in long-read derived metagenome-assembled genomes (MAGs) in sediments

Given the high similarity in the microbial diversity of the sediment samples and to assess the effect of sequencing effort in the representation of species in MAGs, we employed two co-assembly strategies. When we concatenated the metagenomic files chronologically, we observed a gradual increment in the number of recovered MAGs (Fig. S6). Starting with 8 MAGs from the initial single metagenome, we reached 75 MAGs after concatenating the five replicates from sample Mar18 (A1 to A5). We increased the number of recovered MAGs to 92 when the remaining four metagenomes (from Apr18 to Jan19) were included. Similarly, we observed a linear increase in MAGs when concatenating all samples and subsampling to match the Gbps size of the previous strategy. These results highlight the benefits of increasing the sequencing effort to recover MAGs in complex samples when using LR metagenomic approaches (Fig. S6).

All MAGs recovered using both concatenated metagenomic strategies (Table S7) were de-replicated at genomospecies (gspp) level using a 96% average nucleotide identity (ANI) threshold, resulting in the selection of 115 representative gspp MAGs (Table S8). Their genomic completeness ranged between 50% and 98.3% (average 79.2 ± 12%), and 99 encoded an almost complete 16 S rRNA gene sequence. A total of 23 gspp were identified as high-quality MAGs according to MIMAG standards (completeness ≥ 90% and contamination ≤ 5% [73]). The class-level taxonomic classification based on GTDB and SILVA agreed, and both indicated that MAGs were affiliated with the classes *Gammaproteobacteria* (*n* = 55), *Alphaproteobacteria* (*n* = 14), *Acidimicrobiia* (*n* = 17), *Acidobacteriota* class *Thermoanaerobaculia* (*n* = 5), *Bacteroidia* (*n* = 4), *Planctomycetia* (*n* = 3), *Desulfobacteria* (*n* = 3), Gemmatimonadetes (*n* = 3), *Anaerolineae* (*n* = 2), *Nitrospinia* (*n* = 2), *Dehalococcoidia* (*n* = 1), *Cyanobacteriia* (*n* = 1), *Nitrospiria* (*n* = 1), *Phycisphaerae* (*n* = 1), *Entotheonellia* (*n* = 1), *Nitrososphaeria* (*n* = 1) and *Myxococcota* (*n* = 1) (Table S8). The taxonomic classification of the recovered gspp revealed a significant level of novelty, as none of the 115 representative gspp MAGs could be classified at the species level using GTDB. Only five gspp could be assigned to known genera, including *Bythopirellula*, *Breoghania*, *Pseudocolwellia*, and *Tateyamaria*, and *Nitrosopumilus*, while 45 and 26 MAGs were affiliated with well-characterized families and orders, respectively (Table S8).

The combined relative abundance of all MAGs ranged from 7.4 to 11.4% of the total microbial community (in the Sep18 and Mar18_A4 samples, respectively) (Table S8) [Note that the relative abundance of gspp was estimated based on sequencing coverage; then, variations in genome size among not identified species cannot influence our estimations [32]. The observed relative abundances at class level were consistent with those detected using OTUs, with the *Gammaproteobacteria*, *Alphaproteobacteria*, and *Acidimicrobiia* classes being the most abundant (Fig. S7). None of the MAGs independently reached a relative abundance > 0.82%, suggesting a lack of dominance by any single species (Table S8). Interestingly, the order *Woeseiales*, belonging to the *Gammaproteobacteria* class, was the most diverse (15 MAGs representing 8 genera and 2 families) and the most abundant in all metagenomes, averaging 1.3 ± 0.1%. Finally, the relative abundance of the only MAG belonging to the archaeum *Nitrososphaeria* was, on average, 0.3 ± 0.1%.

### Water column microbial diversity compared to sediment

**Fig. 1:**
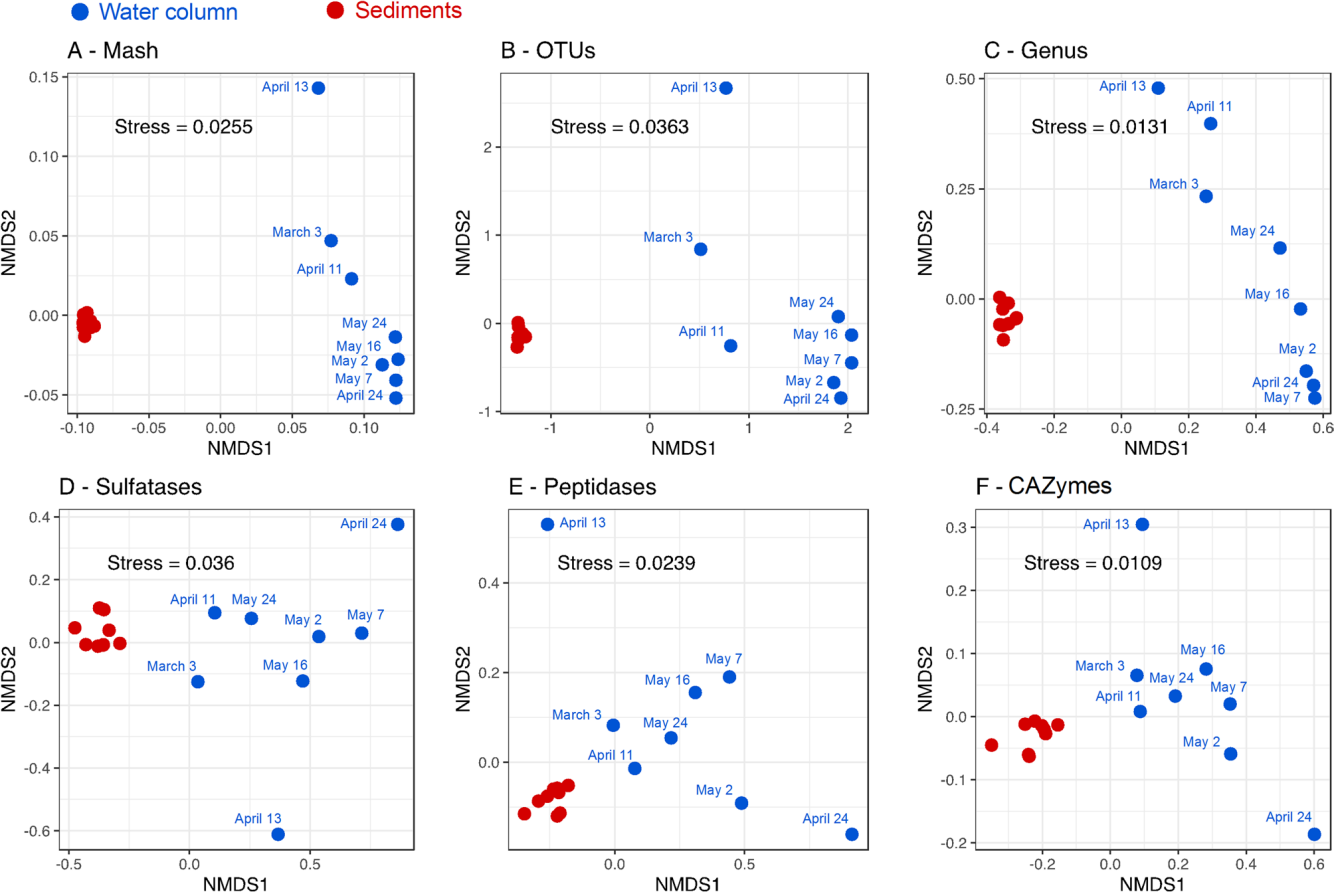
Relatedness between metagenomic samples from sediment (red) and water column (blue) using different approaches: (**A**) Non-metric Multidimensional Scaling (NMDS) analysis based on MASH-based distances; (**B**) NMDS using taxonomic classification of 16 S rRNA genes from raw reads based on OTU_0.98_; (**C**) NMDS using taxonomic classification of raw LRs at genus level; (**D**) NMDS analysis based on gene content encoding for sulfatases; (**E**) peptidases; (**F**) CAZymes

A comparison of sediment and water column LR metagenomes using k-mer-based distances (i.e., MASH) highlighted a statistically significant dissimilarity between the microbial communities and their metabolic gene content (Fig. 1), as supported by a PERMANOVA test (adonis2; R² = 0.615, F = 23.94, *p* = 0.001). This clear separation between the genetic potential of benthic and pelagic microbiomes was also observed when using taxonomic information for contigs (family and genus) and functional profiling depending on CAZymes, peptidases, and sulfatases (Fig. 1). Furthermore, an evident temporal stability was characteristic of sediment microbial communities in all dissimilarity-based analyses. In contrast, microbial populations in the water column were dynamic, as previously observed [55].

To determine the similarities and differences between sediment and water column microbiomes at the species level, we examined the composition of OTUs determined from 16 S rRNA sequences recovered from LR metagenomic datasets. Between March 19 and May 29 of 2018, we identified 993 and 621 OTUs in sediments and water column samples, respectively. When comparing all water column and sediment samples, we identified 276 OTUs shared between both fractions, but only nine OTUs were shared between all samples (Fig. S8).

**Fig. 2:**
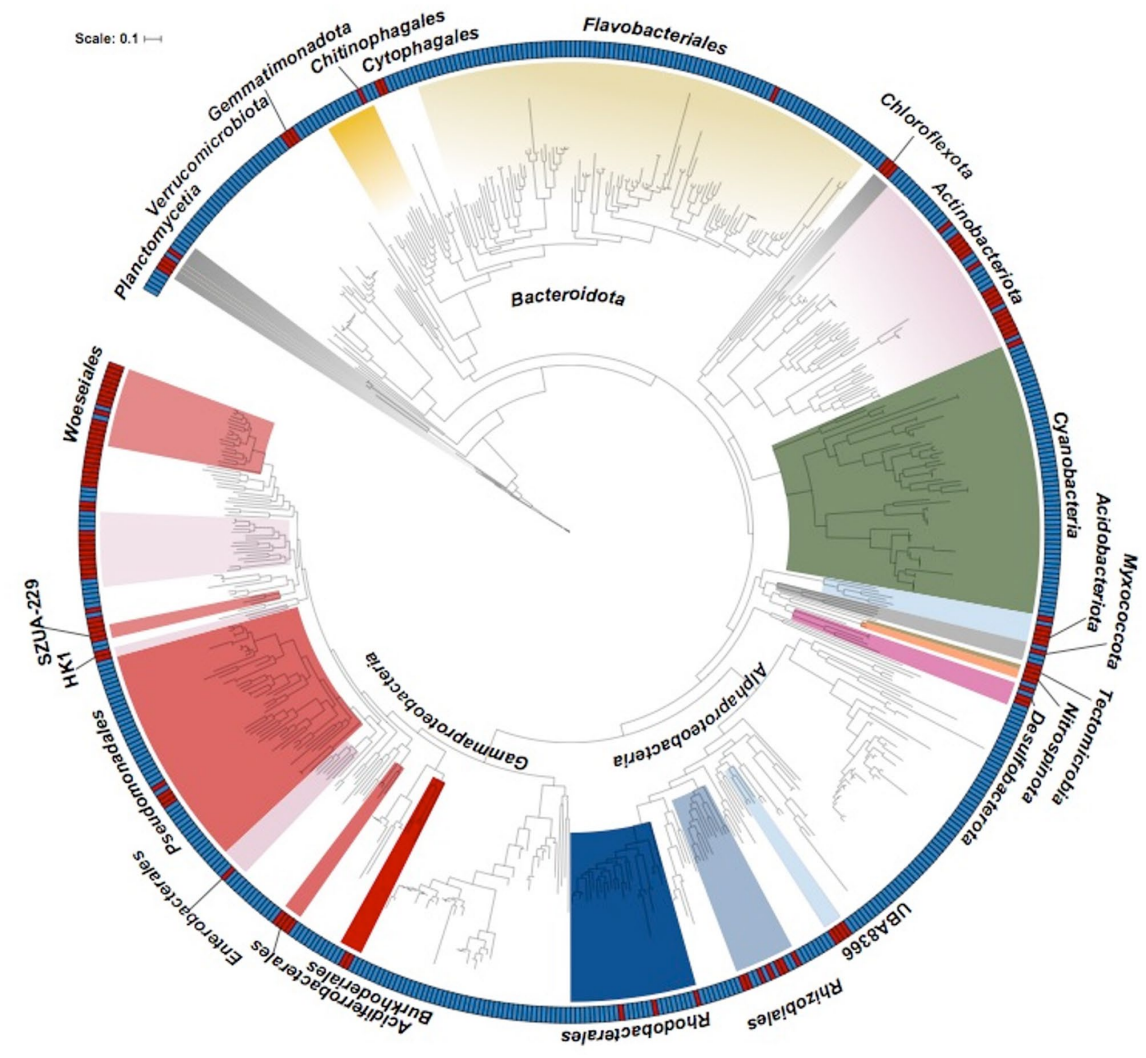
Phylogenetic reconstruction of the MAGs recovered from sediments and water column metagenomes in Helgoland. The maximum-likelihood tree and taxonomic classification were based on the conserved single-copy genes. Class and order names are indicated for MAGs recovered from sediment or water column metagenomic samples. Colored clades indicate the phylum, classes or orders indicated in the outer part of the phylogenetic tree. MAGs recovered from sediment (red) or water column (blue) samples are indicated in the external ring

During the same time frame but at the genomic level, 262 bacterial and 8 archaeal representative gspp (based on MAGs) were recovered from water column metagenomes. These genomes were recovered from three size fractions (0.2-3, 3–10, 10 + µm), thus representing a proxy for free-living and particle-attached fractions, and also the samples spanned different phases of the spring phytoplankton bloom, including an early diatom-dominated phase and a later phase dominated by haptophytes of the genus *Phaeocystis* (Table S9) [55]. The average genome completeness of the MAGs was 82 ± 1.75%, and according to MIMAG standards [73], 111 gspp were classified as high-quality MAGs. All recovered gspp represented for an average of 40.9 ± 6% of the total microbial population across the temporal series of the water column metagenomic dataset [55]. A phylogenetic reconstruction using all bacterial gspp from the sediment and water column revealed significant differences in microbial diversity between the two environments (Fig. 2). While *Gammaproteobacteria* species were recovered from both sediment and water column metagenomes, the order *Woeseiales* was more abundant in sediments and orders *Pseudomonadales* or SAR86 were predominant in the water column. Also, *Alphaproteobacteria* MAGs from sediments were predominantly assigned to *Rhizobiales*, while those from water column samples were related to *Pelagibacterales*. In water column metagenomes, the most diverse phylum was *Bacteroidota* (*n* = 85), particularly within the *Flavobacteriales* order (*n* = 69). In contrast, in sediments, only four MAGs were identified as *Bacteroidota*, two of which were affiliated with *Cytophagales*. *Verrucomicrobiota* and *Patescibacteria* were exclusively recovered from the water column.

Only two MAGs from sediments were identified at the species level in the water column metagenomes. The Desulf_03 MAG shared a 99.2% ANI with the *Desulfocapsaceae* MAG GCA_905479735, and the Acti_15 shared a 98.1% ANI with the *Acidimicrobiia* MAG GCA_905480055. We mapped the short metagenomic reads from water column samples on the sediment-recovered MAGs to further corroborate their presence in water column metagenomic samples. The sediment Desulf_03 MAG had 28x sequencing depth (TAD80) and a 99% sequencing breadth of coverage (i.e., the fraction of genome covered by metagenomic reads). The Acti_15 MAG only had ∼ 4.4x sequencing depth (TAD80) and 86% sequencing breadth of coverage. Interestingly, in the water column, both species were only identified in the particle-attached fractions metagenomes throughout the temporal series, with their maximum relative abundance observed in March and early April, coinciding with the pre- and initial stages of the spring algae bloom (Fig. S9). Acti_15 was more abundant in the sediment metagenomes than Desulf_03, with an average relative abundance of 0.12 ± 0.04% and 0.03 ± 0.01%, respectively (Table S8).

### Organic matter degradation potential in water column vs. sediments

To explore the potential contribution of sediment microbial communities to the degradation of organic matter, we compared the carbohydrate-active enzymes (CAZymes) gene composition, including glycoside hydrolases (GH) as well as peptidases and sulfatases, among the representative MAGs from each gspp identified in both sediment and water column samples. On average, the fraction of genes encoding these polysaccharide degradation genes was lower in sediment MAGs than those from the water column (Fig. S10). Nonetheless, a higher fraction of GH and peptidase genes were encoded in sediment and water column samples for *Bacteroidota* and *Gammaproteobacteria* MAGs, respectively. High sulfatase gene content was characteristic of *Planctomycetota* from the water column and *Bacteroidota* in sediments. While water column populations associated to the spring blooms are characterized for carrying polysaccharide utilization loci (PULs) [2, 6, 12, 14, 15], only 17/115 gspp recovered from sediments carried PULs or CAZyme clusters (Table S10). Here, we define a PUL as a genomic region of ∼ 10 genes, with at least four annotated as CAZymes, alongside transporters, SusCD genes, sulfatases, and/or peptidases in most cases. In sediments, the most commonly detected PULs were associated with the degradation of laminarin (*n* = 14), followed by alginate (*n* = 9) and α-glucan (*n* = 8). The majority of these PULs encoded in sediment MAGs were affiliated with the *Woeseiaceae* family (eleven MAGs; see below), *Bacteroidia* (three MAGs) and one *Acidobacteriota* (family *Thermoanaerobaculia*) MAG.

### Microbial diversity of Woeseiaceae family

**Fig. 3:**
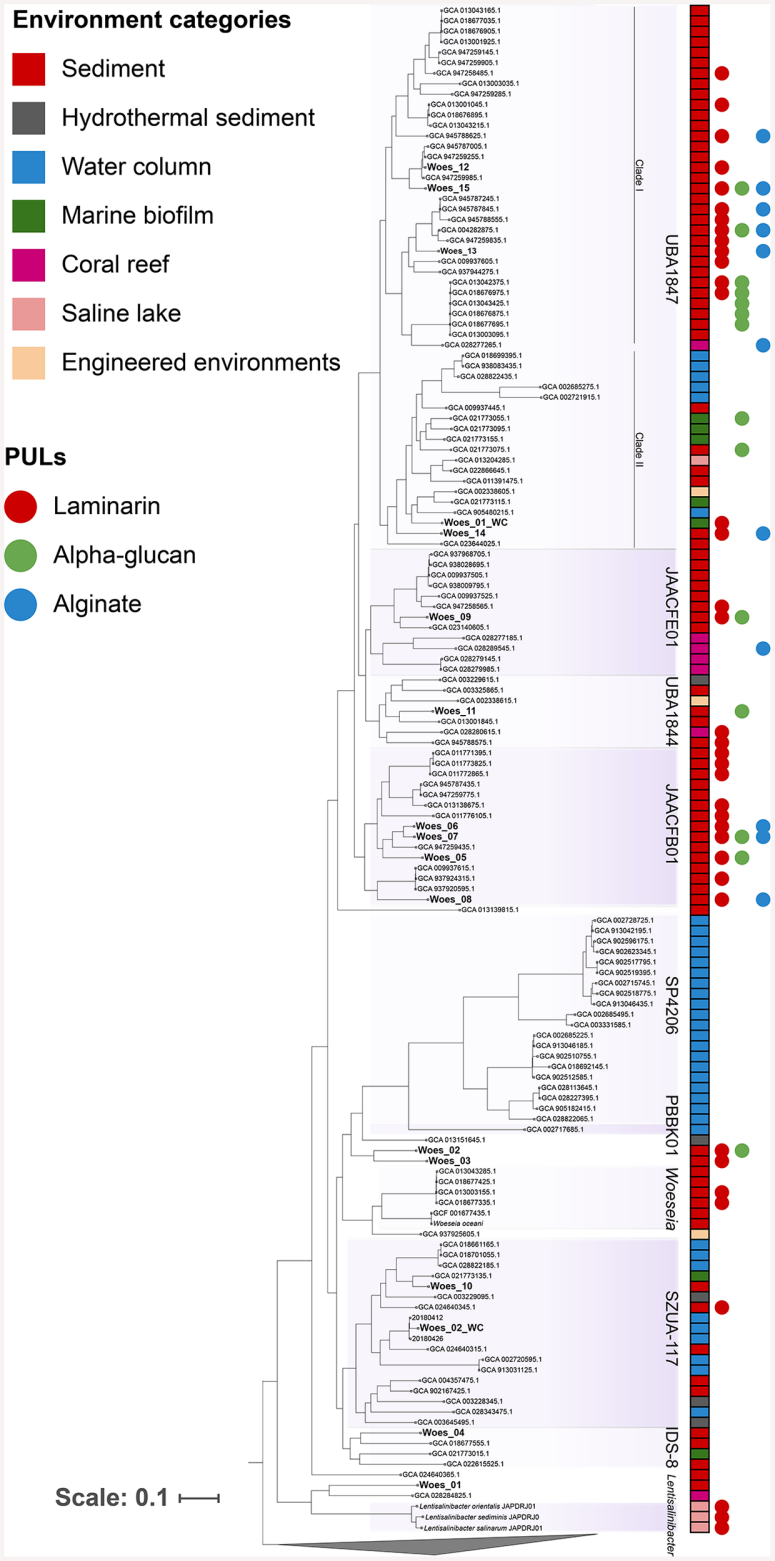
Phylogenetic reconstruction of the *Woeseiaceae* genomes using reference genomes, including MAGs recovered from sediment and water column metagenomes in Helgoland. Genomes marked in bold were retrieved from sediment and water column samples (this study), and others were obtained from NCBI and the Genome Taxonomy database. The colored squares indicate the source of each genome and MAG. The colored circles indicate the presence of laminarin (red dots), alpha-glucan (green), and alginate (blue) PULs. Genus-level classifications and corresponding codes were assigned based on the GTDB taxonomy

Based on the taxonomic classification of unassembled LRs using the Metabuli tool at the family level, *Woeseiaceae* emerged as one of the most abundant families in sediment samples, with an average relative abundance of 2.7 ± 0.3%, in contrast to a significantly lower average of 0.5 ± 0.6% in the water column (Fig. S11), corresponding to a Log_2_ fold-change > 2.5, highlighting a strong enrichment in sediments. Additionally, *Woeseiaceae* represented the most diverse family at species level in the Helgoland sediments, comprising 50 OTUs based on 16 S rRNA gene level and 14 MAGs, with an average completeness of 86 ± 9% and contamination of 2.7 ± 2%. *Woeseiaceae* MAGs encoded the largest number of PUL structures identified in the recovered benthic MAGs. We compared the phylogenetic context of the *Woeseiaceae* genomes recovered in the North Sea to those recovered from different sources (Fig. 3). At first glance, we found that members of different genera were specific to their source. For instance, species of the genera UBA1847 clade I, JAACFE01, UBA1844, JAACFB01, IDS-8 and *Woeseia* were retrieved from sediments, coral reefs or marine biofilms (marked in red, pink and green in Fig. 3, respectively). Contrastingly, species of genera UBA1847 clade II, SZUA-117, PBBK01, and SP4206 were predominantly recovered from water column samples (marked in blue in Fig. 3). Distinctions in genome size between MAGs recovered from sediments and water column were observed (Fig. S12), with sediment MAGs showing a larger genome size (average 3.1 ± 0.8 Mbp) compared to those from the water column (1.7 ± 0.7 Mbp).

To further understand the ecological role of *Woeseiaceae* species in sediments, we compared the MAGs recovered from sediments to those recovered from the water column at the North Sea [55]. In the Helgoland sediments, the Woes_10 (affiliated with SZUA-117) and Woes_08 (JAACFB01) were the most abundant *Woeseiaceae* species with average relative abundance of 0.28 ± 0.11% and 0.23 ± 0.1%, respectively (Fig. S13). No *Woeseiaceae* species originating from sediments were detected in the overlaying water column metagenomes (detection based on read mapping, using a sequencing breadth of coverage cutoff of 20%). The pelagic *Woeseiaceae* MAGs Woes_01_WC (UBA1847 genus) and Woes_02_WC (SZUA-117 genus), were identified in the particle-attached fractions but not in the 0.2–3 μm fraction or sediment samples (based on sequencing depth and breadth; Fig. S14). These two water column MAGs had the highest relative abundance on March 19th in the 3–10 μm, and in April 12th in the 10 + µm fractions (Fig. S14). Thus, when examining all samples recovered from the North Sea, we consistently observed a distinct environmental (i.e., niche) specificity of *Woeseiaceae* species to either sediment or water column samples, which is reflected in their metabolic potential.

### Metabolic potential of Woeseiaceae MAGs

***Woeseiaceae heterotrophy*through*polysaccharide utilization loci (PUL) genes and*peptidases**.

**Fig. 4:**
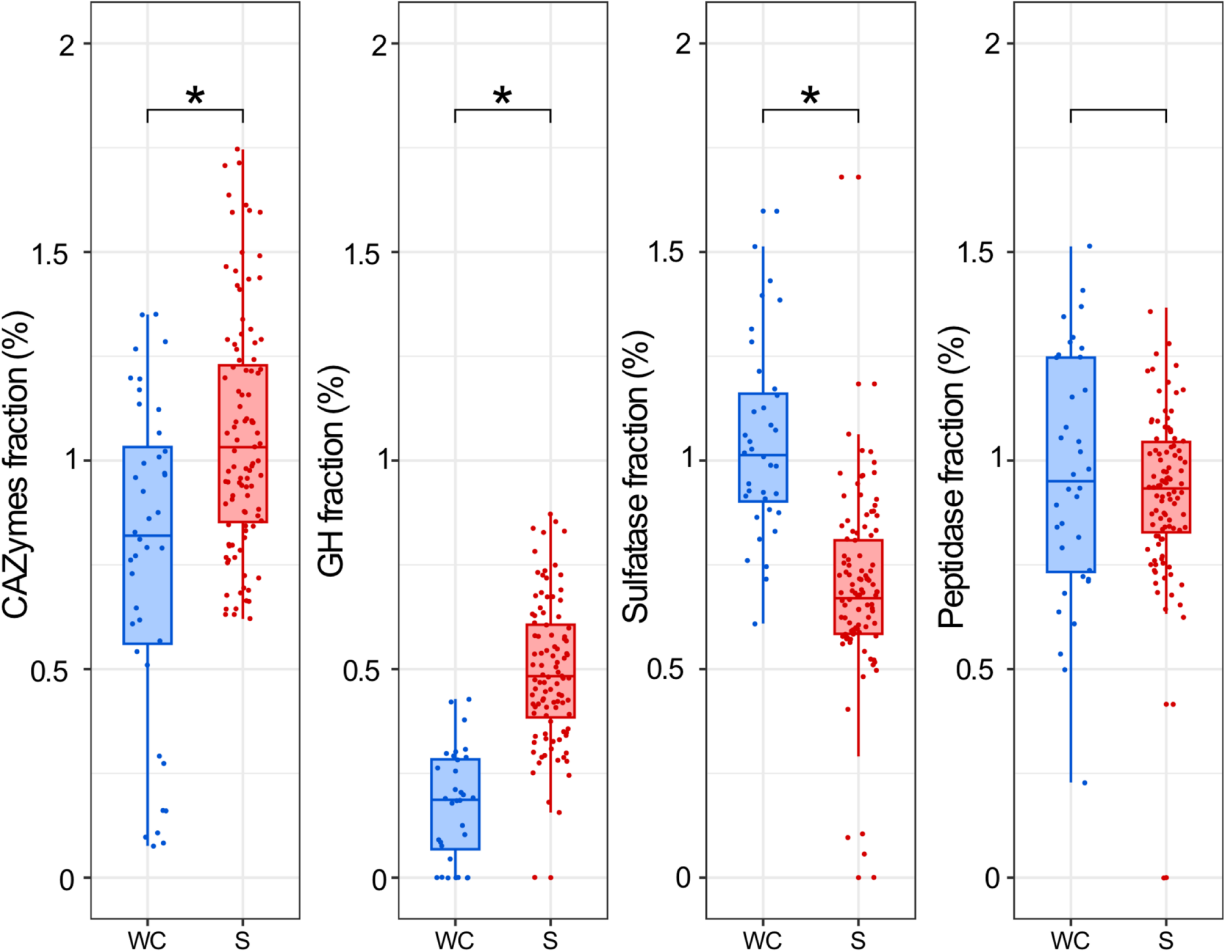
Fraction of genes encoding CAZymes, glycoside hydrolases (GH), sulfatases, and peptidases in *Woeseiaceae* MAGs according to their source. Box plots summarize the fraction of each *Woeseiaceae* MAG dedicated to different functional categories. Each point on the plot represents a single MAG and is colored blue or red according to a water column (WC) or sediment (S) origin, respectively. The asterisk denotes a statistically significant difference determined using the Wilcoxon test (p-value < 0.05)

When examining *Woeseiaceae* genomes recovered from Helgoland and databases (NCBI and GTDB), *Woeseiaceae* genomes encoding PULs were identified only in sediment samples, as well as in particle-attached lifestyles such as marine biofilms and coral reefs (Fig. 3). PULs were not identified in the two *Woeseiaceae* species retrieved from the water column samples (Woes_01_WC and Woes_02_WC). For instance, most of the genomes encoding PULs targeting the degradation of laminarin, α-glucan, and alginate were clustered in the phylogenetic clade containing the genera UBA1847, JAACFE01, UBA1844, and JAACFB01. Moreover, *Woeseiaceae* MAGs obtained from sediment had a higher percentage of genes encoding for CAZymes and GHs when compared to MAGs recovered from the water column (Fig. 4).

We identified that ∼ 79% (11/14) of the recovered *Woeseiaceae* MAGs from Helgoland sediments encoded GHs commonly associated with laminarin PULs, such as GH3, GH16, GH17, GH149, GH13, GH158 and GT51, with seven of these PULs containing a single starch utilization system SusC gene (Fig. S15 and Table S11). It is important to note that these CAZy families are known to act on a variety of substrates beyond laminarin, such as other β-glucans, α-glucans, and related polysaccharides. Six *Woeseiaceae* MAGs encoded genes for α-glucan PULs, characterized by the presence of GH13, GH31, GH97 and GH77, with two PULs containing the SusC gene (Fig. S16). Additionally, six MAGs encoded for alginate PULs, all of them carrying the PL6 and PL7 genes and including the PL17 or PL29 genes, and five PULs encoding the SusC gene (Fig. S17).

MAGs belonging to the *Gammaproteobacteria* class recovered from both water column and sediments encoded the highest fraction of peptidase-encoding genes, in particular, MAGs from sediments belonging to the *Woeseiales* and *Burkholderiales* orders and the *Pseudocolwellia* genus (*Enterobacterales* order) (Fig. S10, Table S11 and S12). Additionally, *Woeseiaceae* MAGs retrieved from the water column had a greater proportion of sulfatases and peptidases, supporting the idea that benthic and pelagic species of this family are specialized to different substrate niches (Fig. 4).

### Niche-specific Woeseiaceae metabolic patterns in different environmental systems

**Fig. 5:**
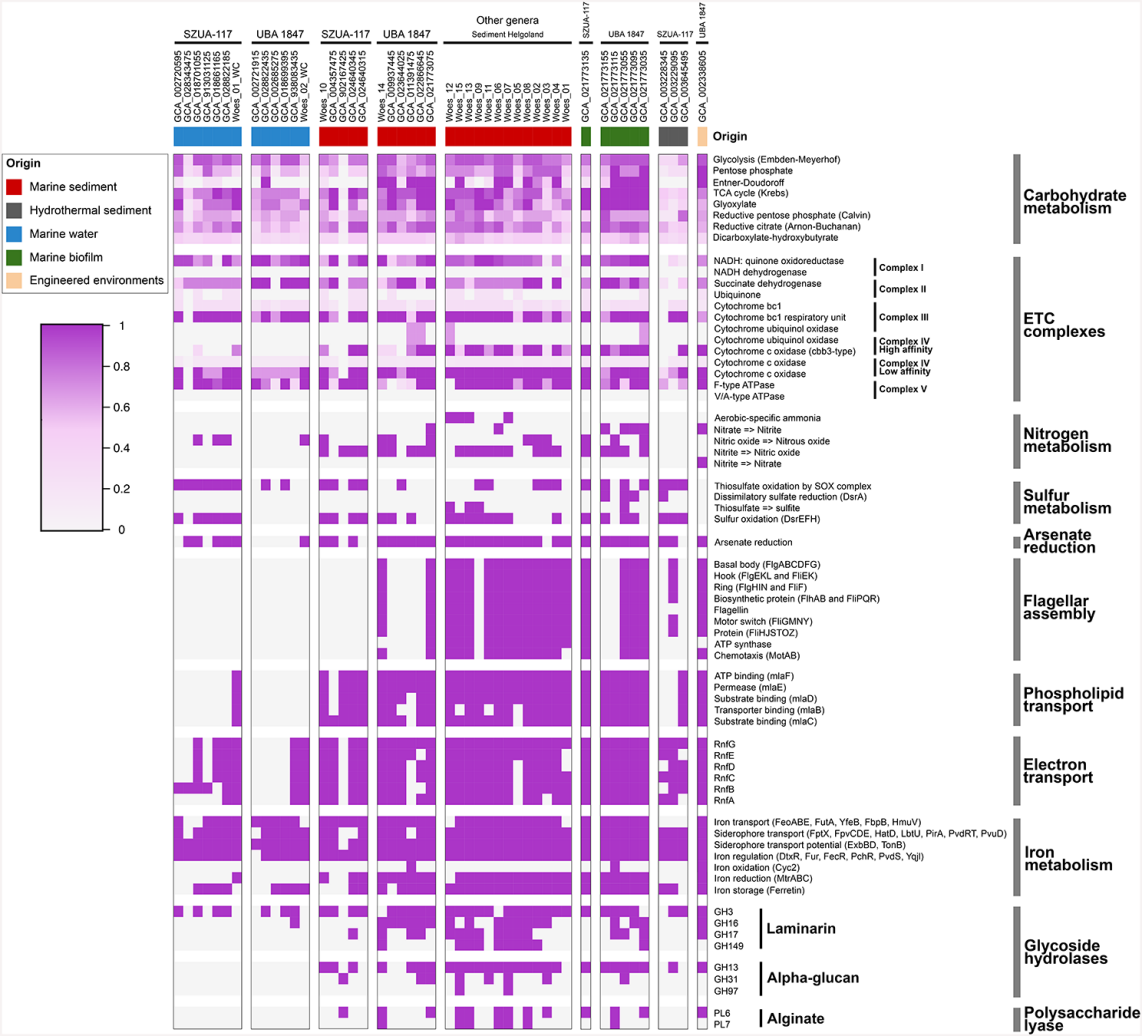
Summary of the metabolic potential for *Woeseiaceae* genomes and MAGs affiliated to genus SZUA-117 and UBA1847. The genomes and MAGs are organized based on their origin

*Woeseiaceae* MAGs affiliated to SZUA-117 and UBA1847 clade II genera were retrieved from the water column and sediment in Helgoland metagenomes, and also from databases. Thus, we sought to examine the genetic components involved in niche-specific metabolic differentiation (Fig. 5). Although some general metabolic pathways such as the TCA cycle and oxidative phosphorylation pathway were detected in all MAGs, source-specific metabolic pathways were also detected. For instance, the Entner-Doudoroff (ED) pathway was exclusively found in genomes from the UBA1847 genus recovered from sediments and marine biofilms. Similarly, genes associated with the electron transport complexes (ETC), such as the cytochrome c oxidase (cbb3-type) of the complex IV were encoded only in MAGs from sediment samples or marine biofilms. Incomplete denitrification pathways were detected in most genomes from sediments, carrying the genetic potential for the nitrate reductase NapAB (reduction of nitrate to nitrite), nitrite reductase NirS/NirK (reduction of nitrite to nitric oxide) and/or the nitric oxide reductase NorBC (reduction of nitric oxide to nitrous oxide). Rubisco (ribulose-bisphosphate carboxylase large chain) required for carbon fixation via the pentose phosphate cycle, was identified only in the sediment MAG Woes_15 suggesting autotrophic metabolism for the minority of the *Woeseiaceae* MAGs recovered (Table S13). The genomic potential for oxidation of reduced sulfur compounds was only detected in sediment and marine biofilm genomes. In terms of phospholipid membrane transport, we observed that the nearly complete Mla (maintenance of outer membrane lipid asymmetry) pathway was predominantly present in genomes from sediment samples.

Genes associated with iron acquisition, especially iron transport, iron gene regulation, and iron storage, were widespread across both water column and sediment-derived *Woeseiaceae* MAGs. Specifically, we detected genes encoding components of heme and ferrous iron transport systems including *hmuV*, *fbpBC*, *feoABE*, *futA1*, and *yfeB*. A diverse set of genes related to siderophore synthesis and transport was identified including *fptX*, *fpvC*, *fpvD*, *fpvG*, the permease *fpvE*, the substrate-binding protein *hatD*. In addition, both tonB-independent (*lbtU)* and tonB-dependent (*pirA)* siderophore receptors were detected, that are typically located in the outer membrane where they bind siderophore-iron complexes. Siderophore export systems were also represented by genes such as *pvdR*, *pvdT*, and the ATP-binding protein *pvuD*. Additionally, energy transduction systems critical for siderophore-mediated iron uptake, including *exbB*, *exbD*, and *tonB* were detected. In terms of iron gene regulation, the MAGs encoded for transcriptional repressors and regulators such as *dtxR* and *fur*, as well as *fecR* and siderophore-specific regulators like *PchR* (pyochelin regulator), *PvdS*, and *YqjI* (regulator for *yqjH*). Genes related to dissimilatory iron reduction, particularly those encoding outer membrane multiheme cytochromes, were predominantly found in sediment-derived *Woeseiaceae* MAGs. These included members of the genus UBA1847 and all MAGs recovered from Helgoland sediments (Fig. 5). Key genes involved in dissimilatory iron reduction included *mtrA*, *mtrB* and *mtrC*, suggesting a functional capacity for extracellular electron transfer to ferric iron. Also, MAGs encoded for genes involved in iron storage, including those encoding ferritin-like domains. Furthermore, most sediment MAGs exhibited a complete set of genes associated with flagellar assembly. The twitching motility genes (PilTIJU) were encoded in all Helgoland *Woeseiaceae* MAGs recovered from sediments and water column. The gene encoding for the tight adherence (TadA) pilus was identified in three MAGs retrieved from Helgoland sediments (Woes_01, Woes_07, and Woes_11), while it was absent in the *Woeseiaceae* MAGs obtained from the water column (Table S13). Overall, the comparative analysis of functional genes among *Woeseiaceae* MAGs retrieved from the water column and sediment indicated a better adaptation of sediment *Woeseiaceae* species to low-oxygen or anoxic conditions.

### Distribution of Woeseiaceae species in North sea sediments

To determine the temporal prevalence of the *Woeseiaceae* gspp recovered in 2018, we examined their presence in Helgoland sediment metagenomes sampled in 2016 at two different depths. Out of the 15 *Woeseiaceae* gspp recovered using LR metagenomics,14 were detected in the superficial sediment layer (0.5 to 2 cm depth) samples from March and April, while all were present in the May sample. Additionally, 12 gspp were detected on a deeper sediment layer (5–6 cm). The Woes12 gspp was the most abundant across the time series and in the 5–6 cm sediment depth (Fig. S18). These results suggest a temporal stability of the *Woeseiaceae* species in Helgoland sediments.

Additionally, we evaluated the spatial distribution of the recovered *Woeseiaceae* gspp in other North Sea sediment samples collected at site Noah-B located North of the Frisian Islands (53.98ºN, 6.51ºE) (for details see [18]). These sediments are approximately 80 km from the Helgoland sampling location and were obtained from two depths (0–2 cm and 2–5 cm). In both sediment layers, Woes12 was detected as the most abundant *Woeseiaceae* species. Additionally, while Woes09 was identified in both layers, Woes15 was detected just in the superficial layer (Fig. S18). These results suggest a wider geographic distribution of three *Woeseiaceae* species in the North Sea.

Our team previously performed a time-series metagenomic examination of sandy surface sediments in Isfjorden (Svalbard) [11]. Although these metagenomic samples represent a broader temporal examination of cold sediments between December 2017 and April 2019, none of the *Woeseiaceae* MAGs recovered at Helgoland were detected in these Isfjorden sediment samples, further supporting the high diversity within this family.

## Discussion

### LRs sequencing considerations in sediment samples

The examination of the metagenomic data recovered here suggested that replicates sequenced from the same DNA and a different DNA extraction of the same sample had the lowest dissimilarity values (based on MASH distance and taxonomic profiles) compared to samples originating from different seasons. Furthermore, the comparison of dissimilarity values between sediment and water column samples, using various taxonomic and functional approaches, confirmed the high seasonal stability of coastal marine sediment microbiomes. Moreover, the LR metagenomic approach allowed the retrieval of an extensive collection of high-quality and full-length 16 S rRNA sequences from the benthic microbial community, which will facilitate further studies interested in designing FISH probes for microscopy-based identification. Despite the high diversity of benthic microbiomes, we could retrieve high-quality MAGs using long-read sequencing and co-assembly. Here, the approach benefited from a high sequencing effort, the temporal stability of microbial communities, and the co-assembly of metagenomic samples for studying complex microbial diversity [74]. The recovered MAGs represented approximately 7–11% of the total microbial community (i.e., the fraction of the reads mapping to the recovered MAGs). This relatively low recovery is primarily due to the high microbial diversity in sediment microbiomes, where many microbial species are present at low abundance, posing a significant challenge to modern sequencing technologies [32]. Nonetheless, the LR metagenomic approach used represents a leap forward for recovering medium to high-quality MAGs from complex environmental samples.

### Temporal stability of sediment microbial communities and interaction with overlaying water column samples

We found a clear phylogenetic separation of MAGs recovered from the sediment and the water column. Together with the presence of specific metabolic pathways, the findings highlight an evolutionary imprint reflected in the genetic specializations and adaptations for bacterial populations recovered from sediments and the overlaying water column (Fig. 2). At the species level, only a small fraction of populations overlapped in the water column and sediment metagenomes (e.g., *Acidimicrobiia* Acti_15 and *Desulfocapsaceae* Desulf_03). We hypothesize that both species originated from the sediment and were subsequently upwelled into the water column, as they were exclusively identified in the particle-attached fractions (3–10 and 10 + µm) and not in the free-living fractions (0.2–3 μm). During spring, the water column is generally higher in turbidity compared to the bloom phase [75], suggesting the resuspension of sediments, and thus explaining the detection of sediment microbial species in the particle-attached water column samples. Moreover, based on the OTU analysis, we identified the archaeon *C.* Nitrosopumilus as the most dominant single species in sediments, likely missed in previous studies relying only on Bacteria domain-specific primers [8]. Members of the phylum *Nitrososphaerota* (formerly Thaumarchaeota) have been identified in oxic sediments in various marine environments, including shallow estuaries, open oceans and deep oceanic crust [77–79]. The microbial composition and temporal stability of benthic microbial assemblages in Helgoland sediment assessed here through deeply sequenced LRs metagenomes match the findings derived from amplicon 16 S rRNA gene sequencing and microscopy cell counts from the same samples [8]. This stability in benthic bacterial communities has been observed in other ecosystems, such as those in Svalbard (Arctic Ocean [11]) or on the island of Sylt (North Sea [80]). While the stability of species abundance is evident in sediments, particularly within the *Woeseiaceae* family, it is noteworthy that these organisms might exist in a latent state for an extended period of time, awaiting opportune conditions (both biotic and/or abiotic) to stimulate their metabolic activity and exploit their ecological niches.

### Metabolic profiling and niche specialization of Woeseiaceae species in sediments and water column ecosystems

*Woeseiaceae* family members have been recognized as highly diverse and abundant microorganisms across sediment environments, including oxic surface coastal sediments, sublittoral, hydrothermal vent, and deep-sea locations [8, 16, 17, 23, 25, 26, 29, 30, 81]. In this study, we observed that all *Woeseiaceae* species were retrieved exclusively from marine environments, including pelagic environments, particle-attached fractions, or marine benthos, but not from freshwater environments. Despite their prevalence in marine sediments, their diversity and metabolic potential remain largely understudied. The metabolic analysis of the *Woeseiaceae* MAGs recovered here supports a chemolithotrophic potential based on the oxidation of hydrogen (presence of oxygen-tolerant [NiFe]-hydrogenase) and of reduced sulfur compounds (encoding SOX gene system) suggested before [23, 25–29]. Moreover, a Rubisco large chain in Woes_15 MAG suggests carbon fixation using the Calvin Benson cycle, also identified in MAG JSS_woes1 recovered from Janssand tidal sediment [29].

Members of the *Woeseiaceae* are mixotrophs. Their heterotrophy is linked to the utilization of polysaccharides and proteins. Here, we found numerous CAZyme genes organized in PULs and peptidases encoding genes [82]. Peptidase genes were enriched in *Woeseiaceae* MAGs from the water column, whereas sediment *Woeseiaceae* MAGs encoded a higher proportion of GH genes (Fig. 4). For example, *Woeseia oceani* has been previously defined through the large diversity of its peptidases, emphasizing their proteolytic potential [16, 23, 29]. The long-read sequencing approach allowed us to recover a PUL organization of CAZyme genes, transporters, and regulators often lost in highly fragmented MAGs recovered using short-read sediment metagenomes. Unlike the PULs frequently found in *Bacteroidota* [83], the PUL structures in *Gammaproteobacteria* were distinguished by the absence of the SusD gene [84]. The lack of typical PUL structures has also been observed in *Verrucomicrobiota* MAGs, which lack both SusCD genes [14]. Then, the sediment *Woeseiaceae* species encoded for PUL structures, which were not encoded in the water column *Woeseiaceae* MAGs, revealing their niche specialization for polysaccharide breakdown and distinguishing them from the water column species that feature more sulfatase and peptidase genes. Although the specific substrates available to water column *Woeseiaceae* remain unclear, our results suggest a higher protein content compared to sediments, which may explain the enrichment in peptidase and sulfatase genes in planktonic Woeseiaceae MAGs. In contrast, sediment *Woeseiaceae* MAGs encoded more glycoside hydrolases, possibly reflecting an adaptation to diverse and often partially degraded polysaccharides that circulate and partially accumulate in benthic environments, including laminarin from mainly benthic diatoms or more recalcitrant polysaccharides that reach the sediment after partial processing in the water column. Although PULs were present in the MAGs and the samples were collected a few days after the chlorophyll *a* peak in the water column and sediments during the phytoplankton spring bloom [8, 55], relative abundance of benthic *Woeseiaceae* did not increase. The stability suggests that other carbon sources than fresh material (e.g., laminarin), grazing, or viral lysis control the growth of the *Woeseiaceae* members.

The phylogenetic reconstruction indicated a differential representation of *Woeseiaceae* genera and species across environmental categories (Fig. 3). Most *Woeseiaceae* species seem attached to surfaces, such as sediment, biofilms, or particles. Species retrieved from pelagic environments were only affiliated with the genera UBA1847 clade II, SZUA-117, and SP4206, indicating an ecological niche specialization. This strong correlation between phylogenetic *Woeseiales* lineages and their source was previously observed by an analysis based on 16 S rRNA genes retrieved from the SILVA 128 database [23].

In the sediment and water column samples from Helgoland, we retrieved MAGs representing *Woeseiaceae* species affiliated with the UBA1847 clade II and SZUA-117 genera, and an evident metabolic divergence was observed depending on their source (Fig. 5). Most MAGs recovered from sediments encoded a complete or almost complete ED pathway, which was not detected in water-column MAGs. The ED pathway provides sediment *Woeseiaceae* species with a versatile metabolism for using glucose under oxic and/or anoxic conditions. Compared to Embden-Meyerhof-Parnas pathway, the ED requires fewer enzymatic steps, enhancing metabolic efficiency and avoid the production of reactive oxygen species (ROS) under anoxic conditions [85, 86]. Moreover, the sediment MAGs encoded the high-affinity complex IV of the ETC, including the cytochrome c oxidase cbb3-type, indicating their potential to respire under low-oxygen or anoxic conditions, and also to perform nitrate/nitrite respiration, depending on prevailing environmental conditions [87]. Under anoxic or low-oxygen conditions, gram-negative bacteria such as *Woeseiaceae* may experience stress that could disrupt the lipid asymmetry of the outer membrane. We observed that the MAGs recovered from sediments encoded the Mla system, which helps restore and maintain this asymmetry by removing excess phospholipids from the outer membrane and transporting them back to the inner membrane [88].

Genes for iron acquisition, iron gene regulation systems, and iron storage were found in *Woeseiaceae* genomes across both, water column and sediment (Fig. 5) supporting that iron limitation is a common selective pressure in marine environments. The prevalence of siderophore transport and multiheme cytochromes, along with the presence of TonB-dependent receptors, indicates an active strategy for scavenging under iron-limiting conditions, an adaptation expected in both pelagic and benthic environments. In contrast, putative genes associated with dissimilatory iron reduction, such as genes for transmembrane electron transfer *mtrCAB* [72], were predominantly found in sediment-derived MAGs, particularly in members of UBA1847 and in all sediment MAGs from Helgoland. These findings suggest a niche-specific adaptation in sediment-dwelling bacteria, which often exploit insoluble iron or manganese oxides as electron acceptors under anoxic conditions [89, 90]. Recently, Karačić and colleagues [90] briefly reported also the potential for iron reduction in *Woeseiaceae* from biofilms on concrete in the Oslofjord subsea tunnel, based on the detection of *mtrCAB*. Another niche-specific adaptation in sediment-derived *Woeseiaceae* MAGs is the presence of flagella and the tight adherence (tad) pilus genes that may promote surface adhesion and enhance colonization and biofilm formation [91–93]. Moreover, all *Woeseiaceae* MAGs, whether from pelagic or benthic environments, encoded for twitching motility genes (type IV pili) that facilitate cell-cell and cell-surface adhesion [94].

## Conclusions

Despite the challenges in assembling metagenomic reads from highly diverse samples such as sediments, the use of a co-assembly approach of LR metagenomes was successful for recovering high-quality MAGs. The approach benefited from the use of LR metagenomics, the high sequencing effort, and the temporal stability of microbial communities. Due to the high microbial diversity in marine sediments, even deep sequencing of whole sequencing runs per sample was not sufficient to cover the majority of the community. We only retrieved ∼ 600 to 900 OTU_0.98_ compared to several thousands of OTUs retrieved by amplicon sequencing [8]. Furthermore, only 11% of the metagenome was represented in the MAGs. Further increase in sequencing effort would help to increase coverage. Considering the high cost of sequencing required to recover high quality MAGs, however, a reasonable alternative would be to analyze microbial diversity and metabolism using raw reads. In this study, using unassembled reads and MAGs we observed characteristic genetic and taxonomic patterns in pelagic and benthic microbiomes.

Detailed comparison of pelagic and benthic microbiomes offered new insights into different lifestyles and substrates utilization. Specifically, *Woeseiaceae* could serve as a model clade to study adaptations and niche differentiation of taxonomically related benthic and pelagic microbiomes.

Both *Woeseiaceae* communities preferentially show an attached lifestyle, either firmly attached to sand grains or attached to particles in the water column. They differed, however, in their gene repertories for degradation of protein-rich and sulfated organic matter or algal derived polysaccharides, and also in iron reduction, highlighting niche-specific strategies for coping with iron availability [95]. Altogether, this approach revealed contrasting adaptations of taxonomically related species thriving in open waters and sediments.

## Electronic supplementary material

Below is the link to the electronic supplementary material.

Supplementary Material 1

Supplementary Material 2

## Data Availability

The metagenomes and MAGs generated in this study were deposited under the European Nucleotide Archive (ENA) accession code PRJEB64856, and accession run codes from ERR11809028 to ERR11809036 (Table S1). The metagenomes and MAGs from the three water column fractions are available under the accession code PRJEB38290 [55]. The 16 S rRNA sequences recovered from PacBio raw reads dataset are available in doi:10.6084/m9.figshare.25577322.
