# Supplementary material for: Comparative metagenomics indicates metabolic niche differentiation of benthic and planktonic Woeseiaceae
Source: Environ Microbiome. 2025 Jun 17;20:74. doi: 10.1186/s40793-025-00732-3 (PMC12175321; doi:10.1186/s40793-025-00732-3)
Supplement: Supplementary file 1 — Supplementary Material 1 [file 40793_2025_732_MOESM1_ESM.pdf]

Supplemental Information for article:

Comparative metagenomics indicates metabolic niche differentiation of benthic and planktonic *Woeseiaceae*

Tomeu Viver[1*], Katrin Knittel[1], Rudolf Amann[1], Luis H. Orellana[1*]

Affiliations

[1] Department of Molecular Ecology, Max Planck Institute for Marine Microbiology, Bremen, Germany.

*Correspondence:

Tomeu Viver

bviver@mpi-bremen.de

Luis H. Orellana

lorellan@mpi-bremen.de.

Figure S1. Relatedness between all metagenomic samples recovered. The Non-metric Multidimensional Scaling (NMDS) analysis is based on MASH-based distances calculated between all pairs of metagenomic reads.

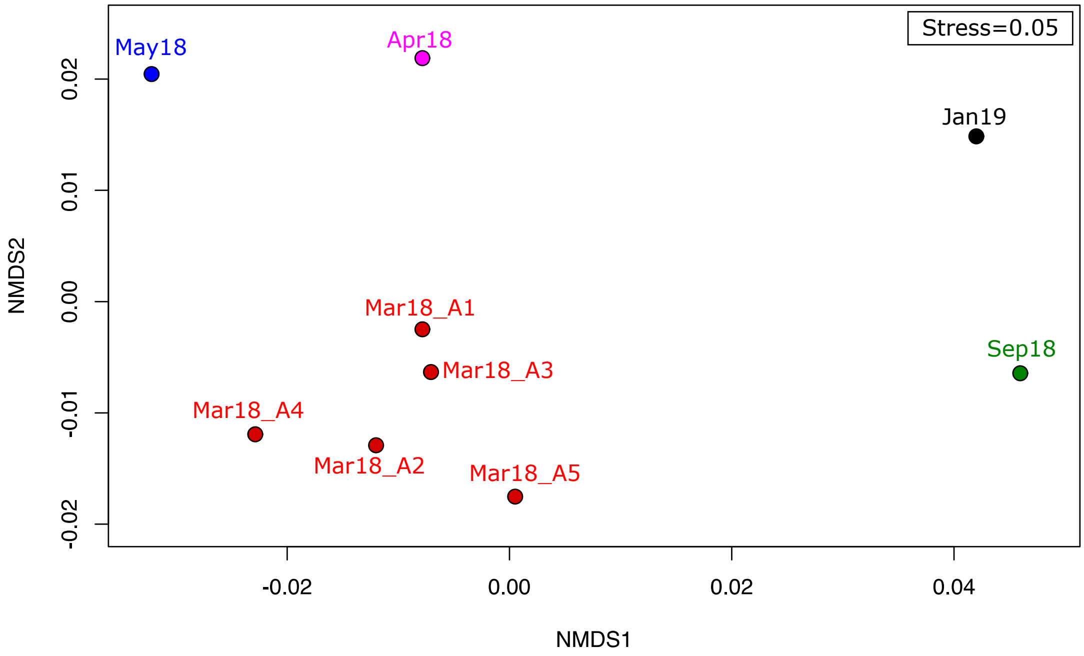

Figure S2. Rarefaction curves based on Bacteria and Archaea OTUs classification of all metagenomic samples. The x-axis denotes the number of 16S rRNA sequences recovered from each metagenomic dataset, while the y-axis corresponds to the number of OTUs (Richness). (A) Rarefaction curve for each individual metagenome. (B) Rarefaction curves for combined datasets: one for the five Mar18 samples (Mar18_Combined_dataset) and one for all metagenomes combined (ALL_Combined_dataset). Singleton and doubleton OTUs were removed from the analysis.

A

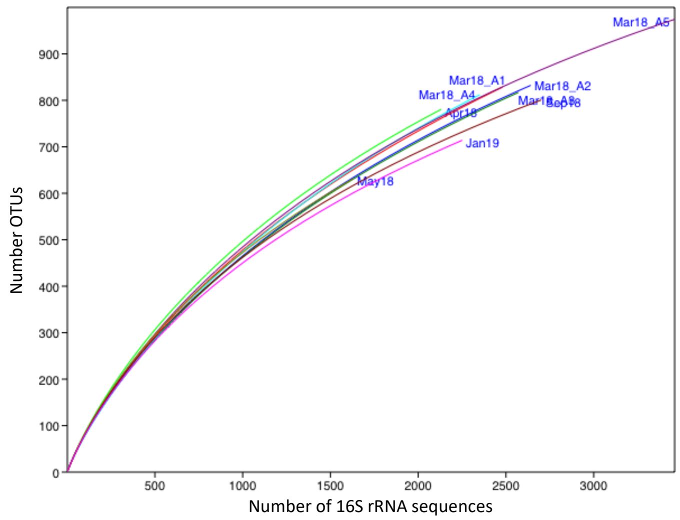

B

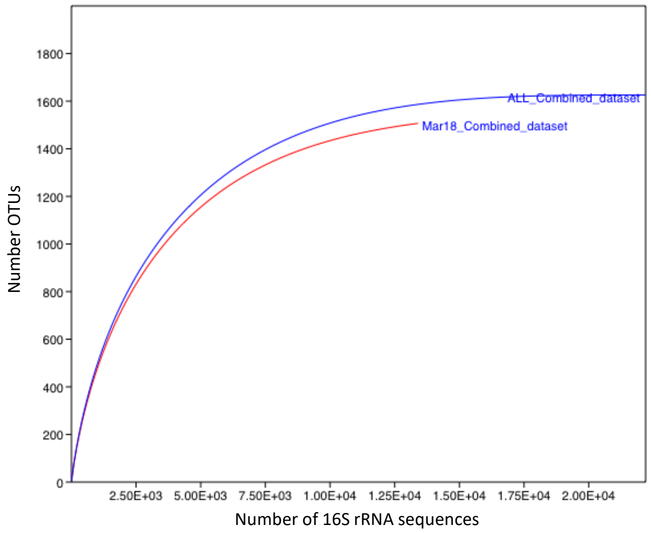

Figure S3. Taxonomic distribution and relative abundance of bacterial and archaeal families and genera from Helgoland sediments derived from recovered 16S rRNA sequences from metagenomes. Taxonomic classification is based on the SILVA SSU138.1 NR99 database. The circle diameter indicates the relative abundance (16S rRNA read frequencies) and color of the sampling season.

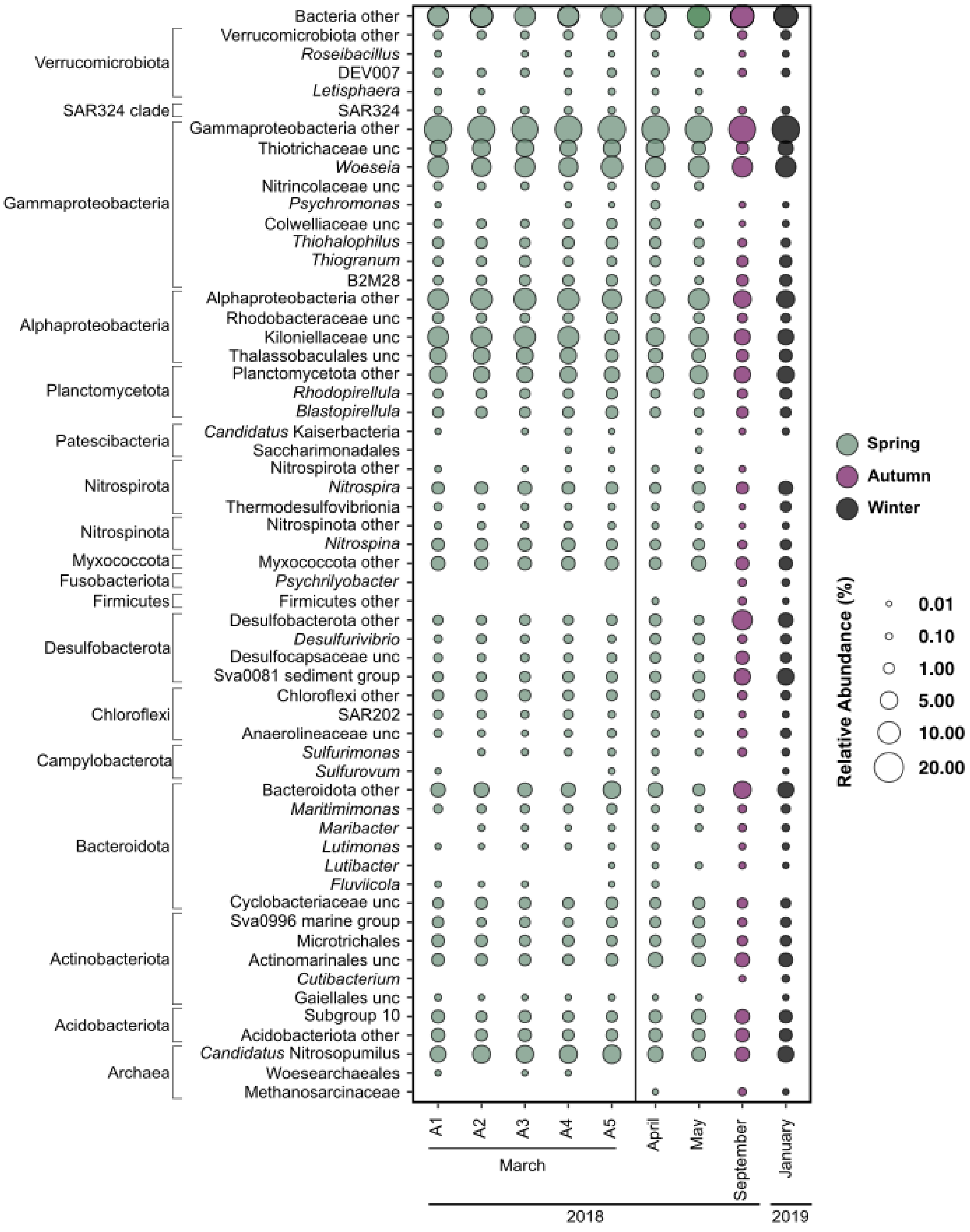

Figure S4. Comparison of **relative abundance values for the most abundant class and order levels in Helgoland sediments.** The results presented here compare the abundances determined using**:** (i) full-length 16S rRNA gene sequences from unassembled PacBio long reads and taxonomic classification based on OTUs, (ii) the taxonomic classification of unassembled metagenomic data reads based on sequence similarity (yellow) and (iii) 16S rRNA amplification approach reported in Miksch et al., 2021 (grey).

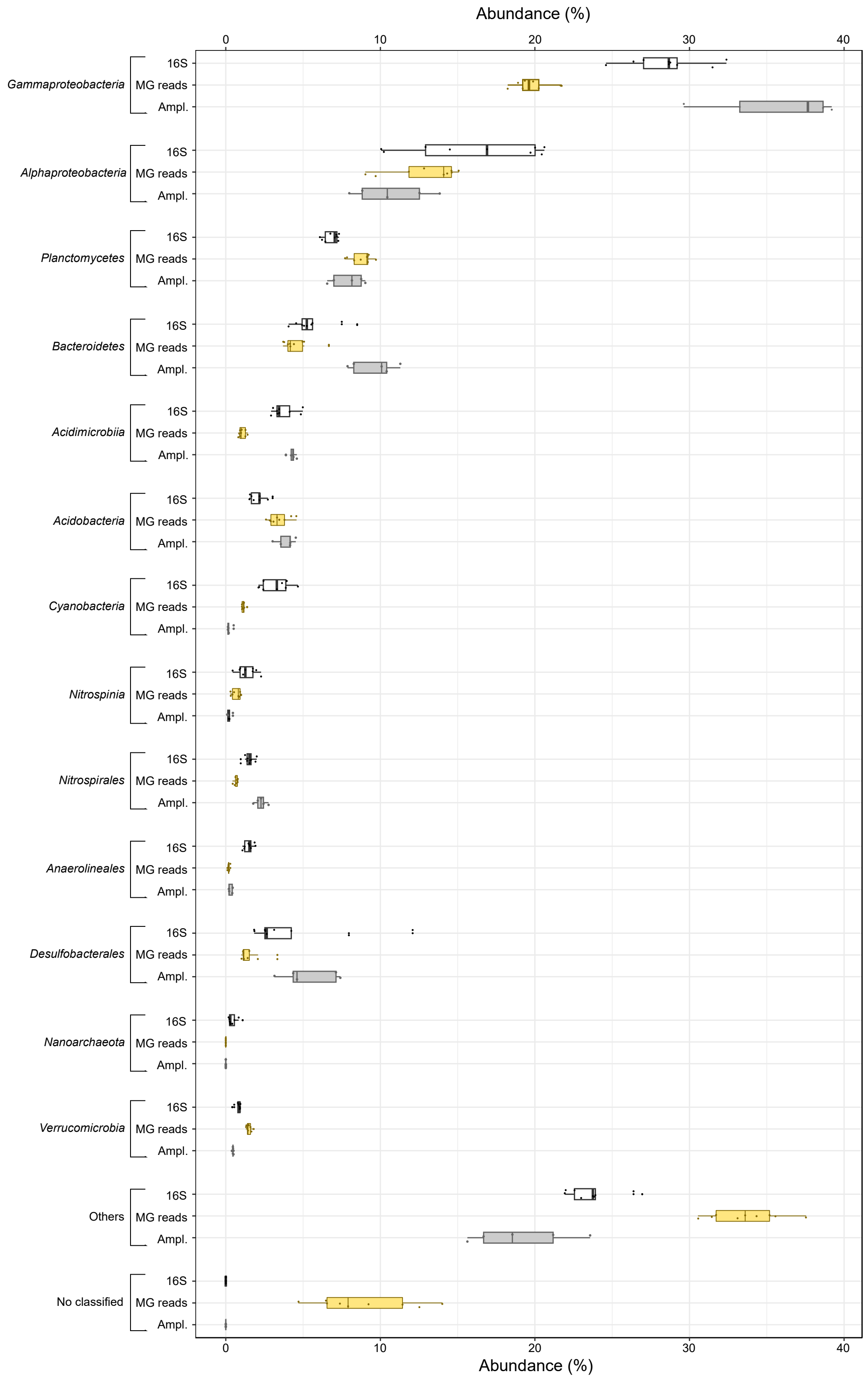

Figure S5. Comparison of relative abundances determined using metagenomic and amplicon sequencing for sediment samples. Comparison of the taxonomic distribution and relative abundances of bacterial and archaeal families and genera reported in Figure S3 using 16S rRNA gene sequences extracted from unassembled LRs and from amplicon sequencing previously reported (Miksch et al., 2021). Statistical significance was determined using the Wilcoxon test (p-value <0.05).

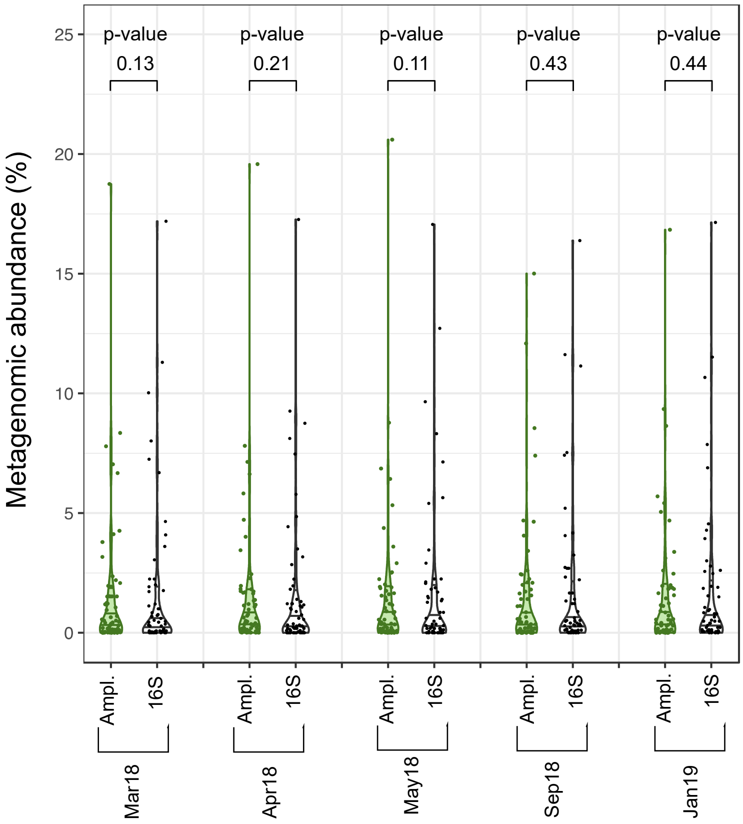

Figure S6. Co-assembled long-read metagenomes increase the recovery of medium and high-quality MAGs from sediment metagenomic samples. MAGs recovered after consecutive co-assembly of metagenomic samples, following the temporal scale order (represented in black line), and MAGs recovered through subsampling of all concatenated metagenomes (discontinuous grey line).

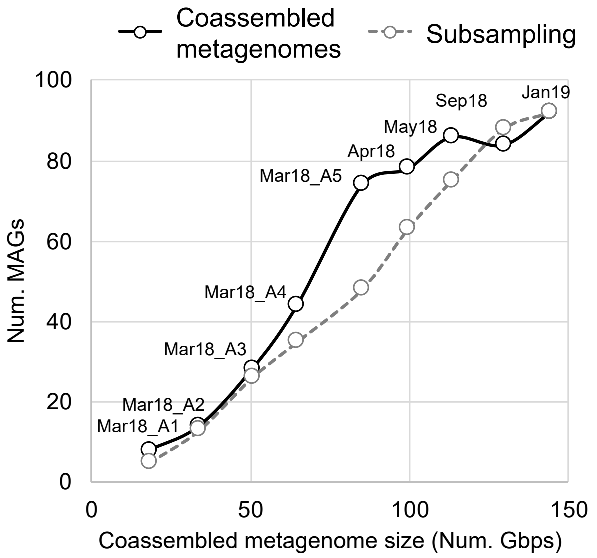

Figure S7. Relative abundance of MAGs, grouped at class level, retrieved from sediment samples. Note that from sample Mar18, five replicates from the sample were sequenced (A1 to A5).

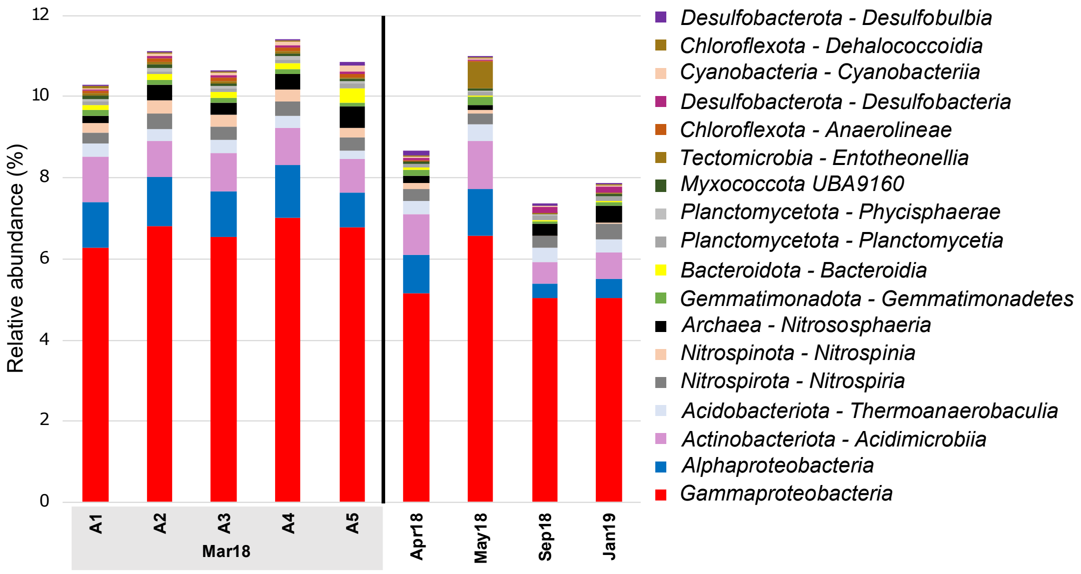

Figure S8. Comparison of OTUs recovered from sediment and water column metagenomic samples collected between March 19 and May 29 of 2018. Sediment and water column samples were collected: in March, sediment was sampled on the 7th and water column on the 20th; in April, sediment on the 17th and water column on the 13th; and in May, sediment on the 15th and water column on the 16th.

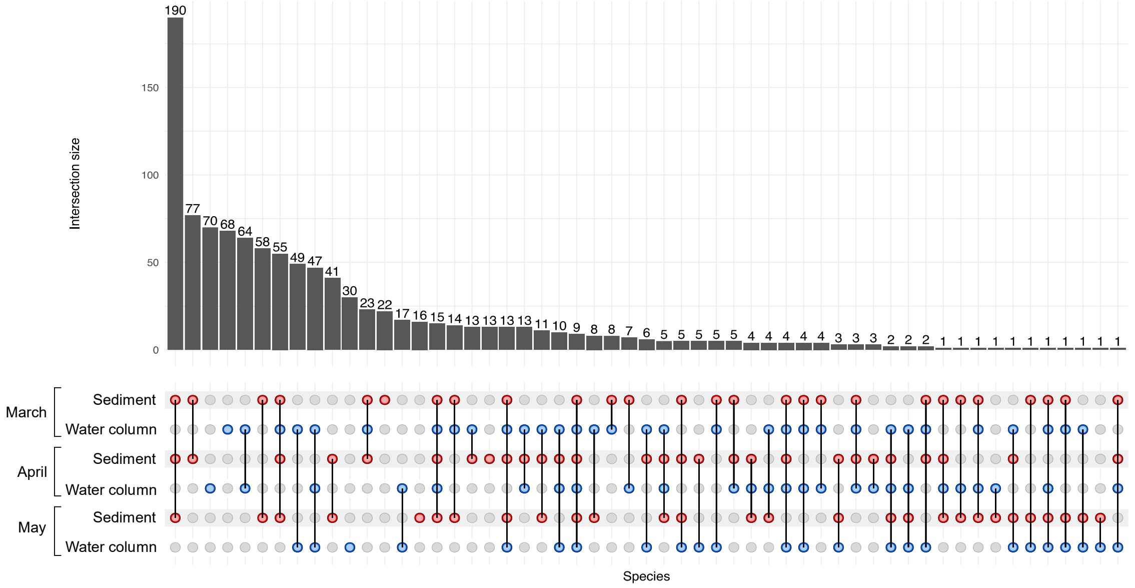

Figure S9. Relative abundance of the Desulf_03 and Acti_15 MAGs, affiliated to the family *Desulfocapsaceae* and class *Acidimicrobiia*, respectively. Their relative abundance was analyzed in the three distinct filtered fractions of the water column samples in 2018 (0.2–3 μm, 3–10 μm and >10 μm fractions) during three months. In samples from 2018-03-19 and 2018-04-12 and the 3–10 μm and >10 μm fractions, the *Desulfocapsaceae* MAG was identified with a sequencing breath (i.e., the fraction of the genome covered by metagenomic reads) >0.96. The *Acidimicrobiia* MAG had a sequencing breath >0.84 in the same samples.

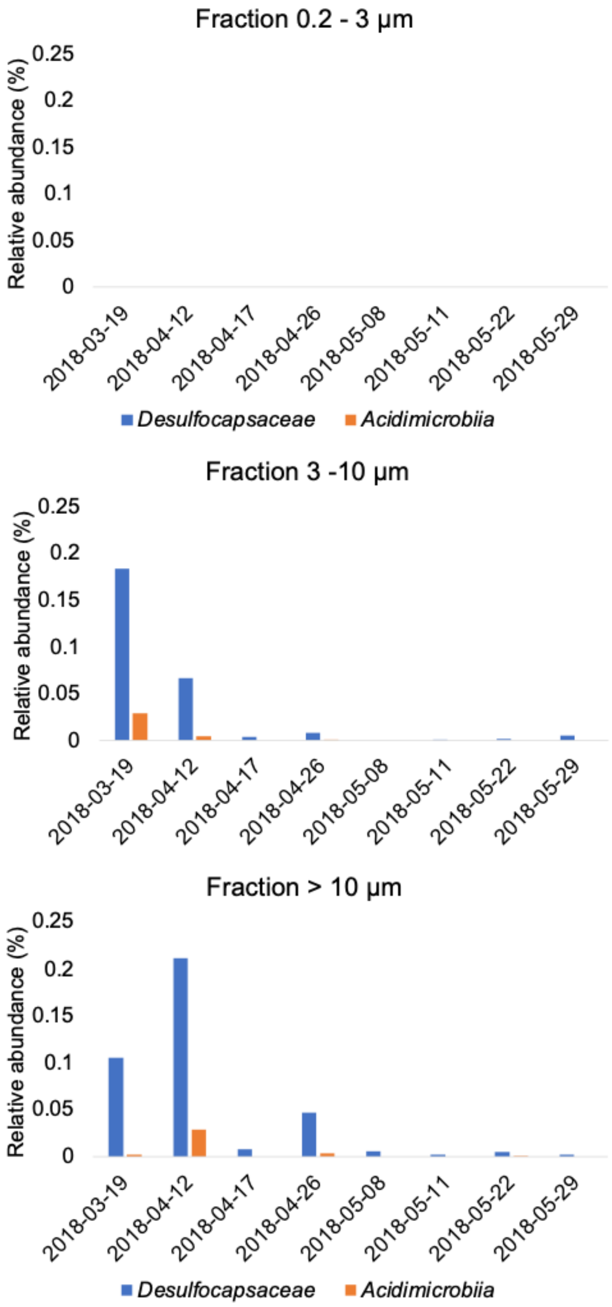

Figure S10. Fraction of genes encoding CAZymes, Glycoside hydrolases (GH), sulfatases, and peptidases in MAGs retrieved from the water column (blue) and sediment samples (red). Every point on the graph represents a single MAG, classified at the class level based on the phylogenetic reconstruction using the marker genes implemented in GTDB-tk tool.

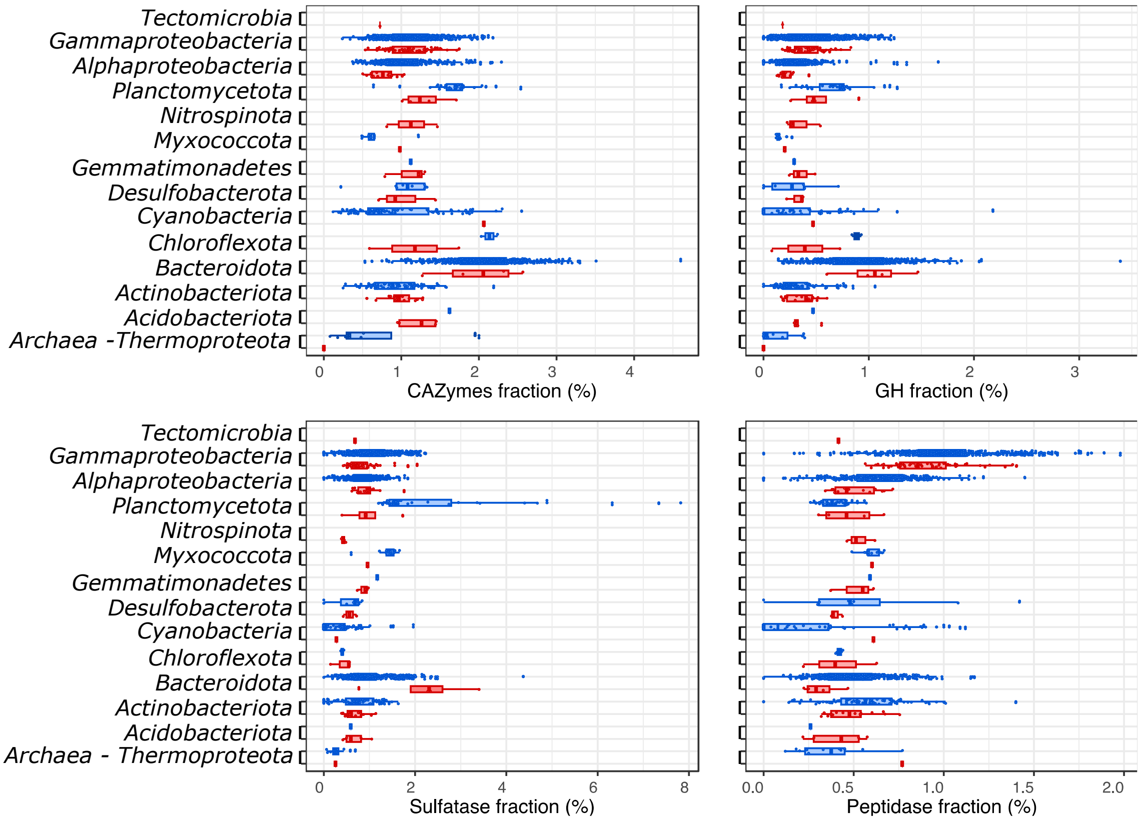

Figure S11: Comparison of family-level relative abundance in long read metagenomic data from Helgoland sediment and water column samples. (A) The ratio of average relative abundance in sediment samples to average relative abundance in water column samples is presented as log2-fold change. Families with log2-fold change values > 1 are more abundant in sediments (red), while those with values <1 are more abundant in the water column (blue). Families with similar abundances in both environments are indicated by gray bars. (B) Average relative abundance of families in sediment samples. (C) Average relative abundance of families in water column samples.

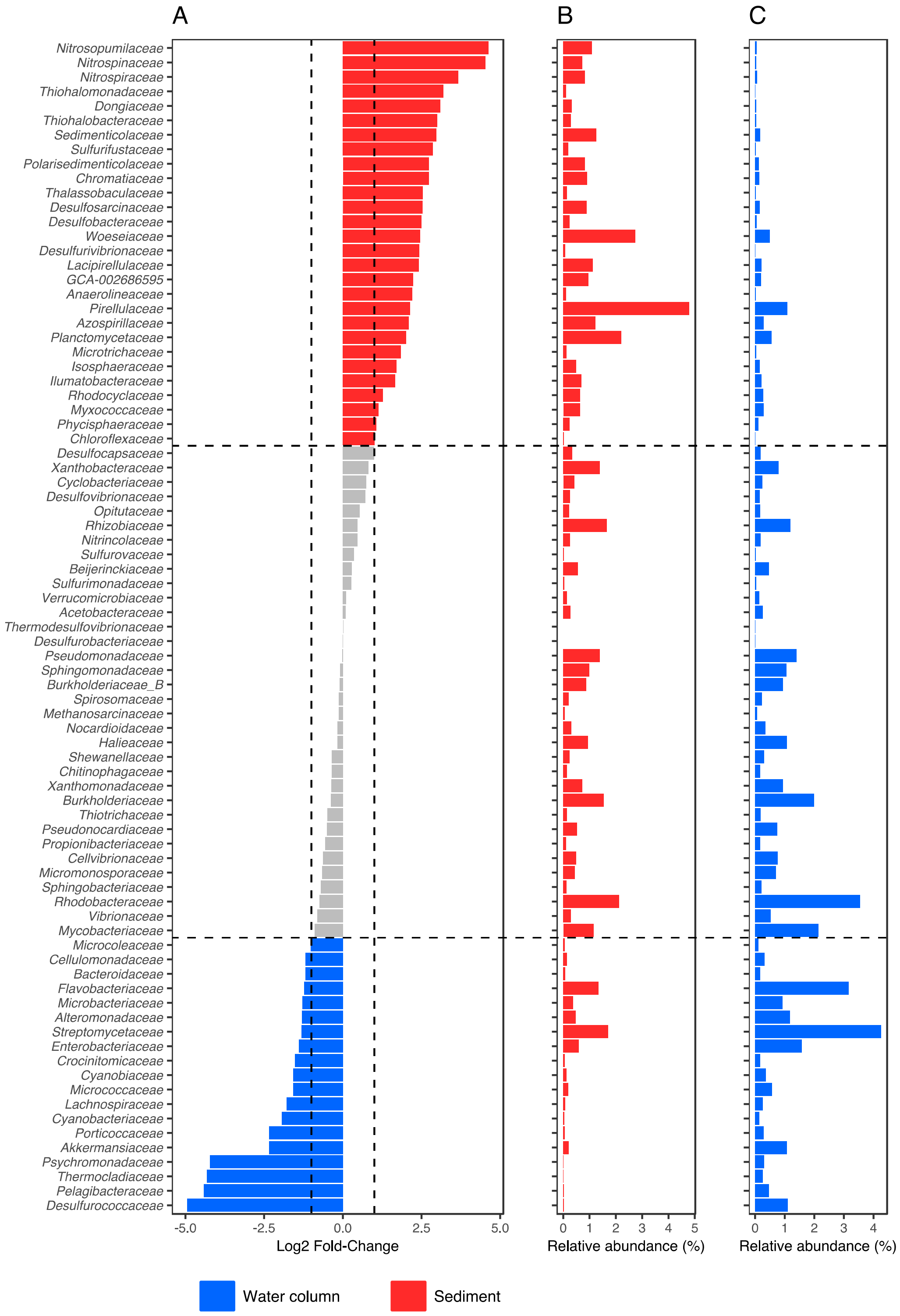

Figure S12. Genome size of the *Woeseiaceae* MAGs classified depending on their water column (WC) or sediment (S) origin. Every point on the graph represents a single MAG and is color-coded based on the environment categories from which it was retrieved. The asterisk denotes a statistically significant difference determined by the Wilcoxon test (p-value < 0.05).

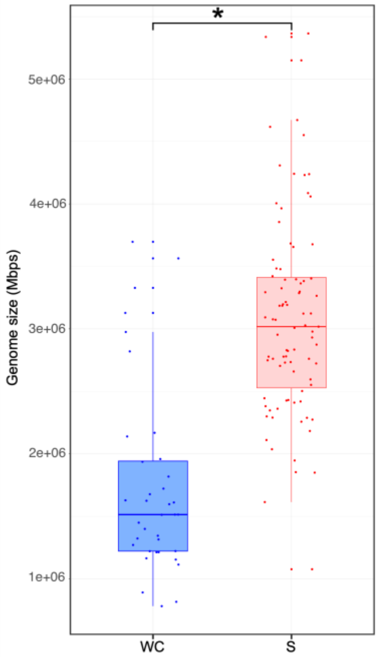

Figure S13. Relative abundance of *Woeseia* MAGs in the sediment samples. The y-axis represents the average abundance of each MAG in the temporal series in sediment metagenomes and its standard deviation.

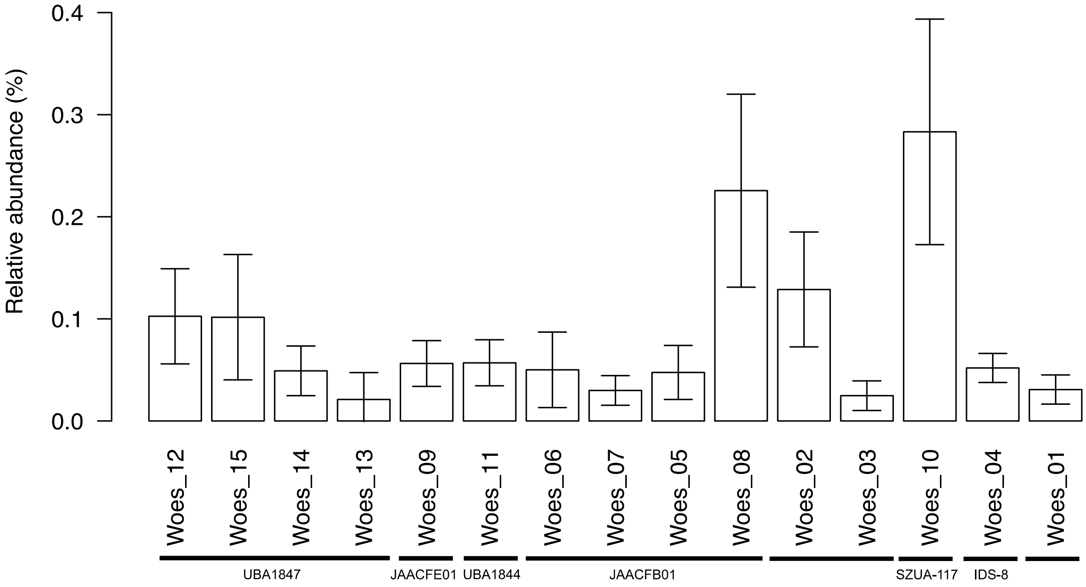

Figure S14: Relative abundance of the *Woeseiaceae* MAGs retrieved from water column metagenomes. Their relative abundance was analyzed in the three distinct filtered fractions of the water column samples in 2018 (fractions 0.2–3 µm, 3–10 µm and >10 µm) and in the sediment samples.

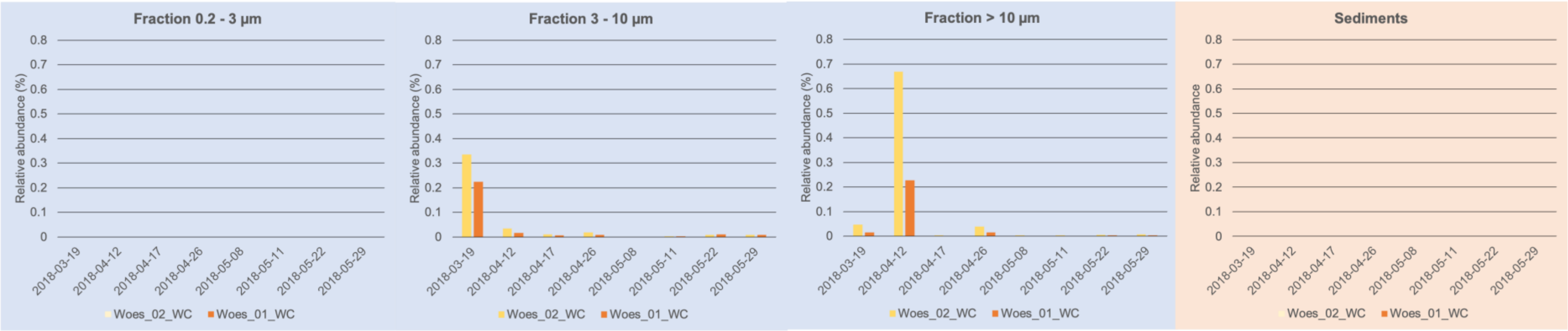

Figure S15: Genetic organization of the laminarin PULs encoded by *Woeseiaceae* MAGs.

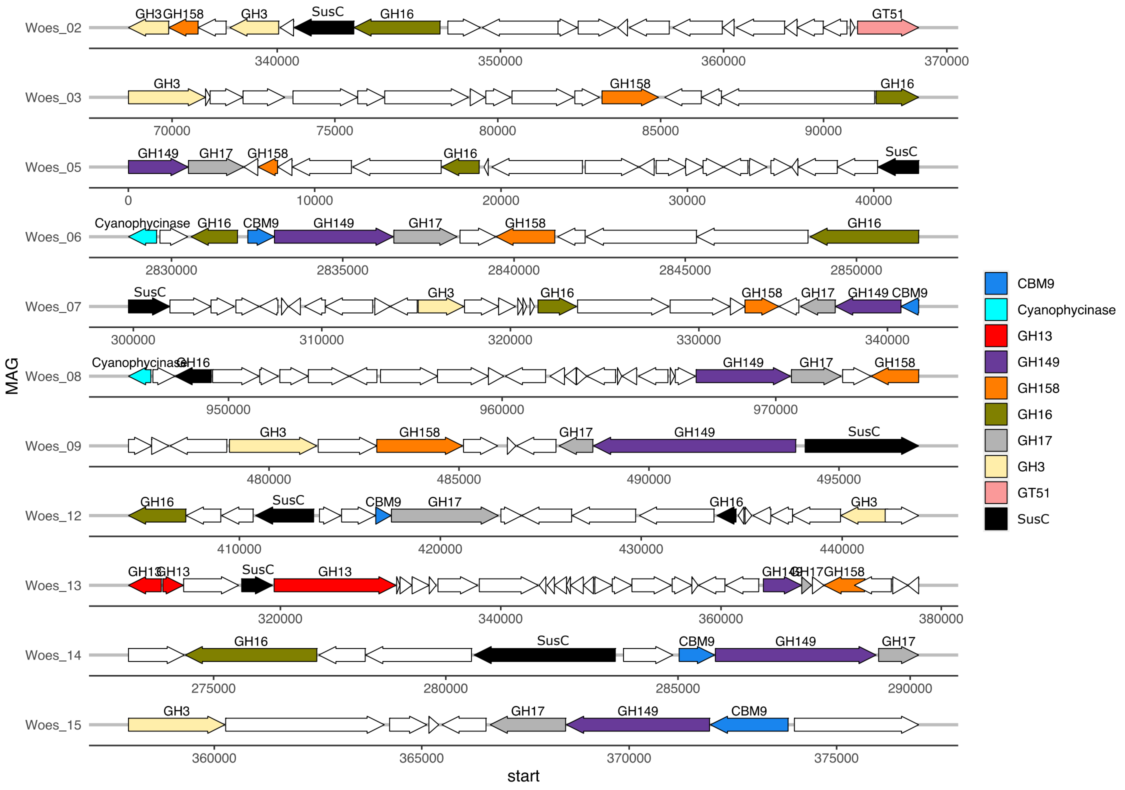

Figure S16: Genetic organization of the alpha-glucan PULs encoded by *Woeseiaceae* MAGs.

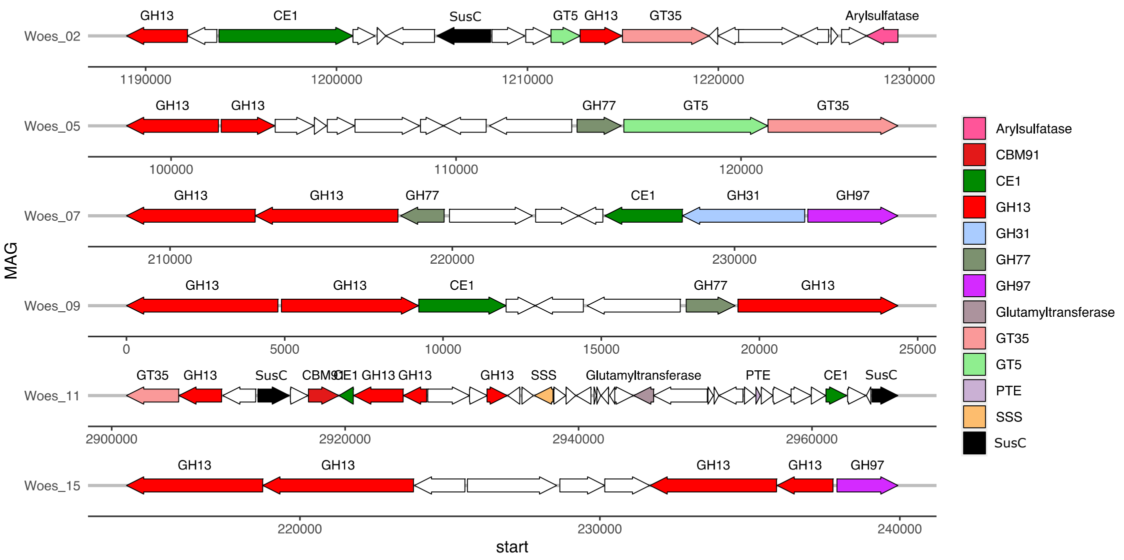

Figure S17: Genetic organization of the alginate PULs encoded by *Woeseiaceae* MAGs.

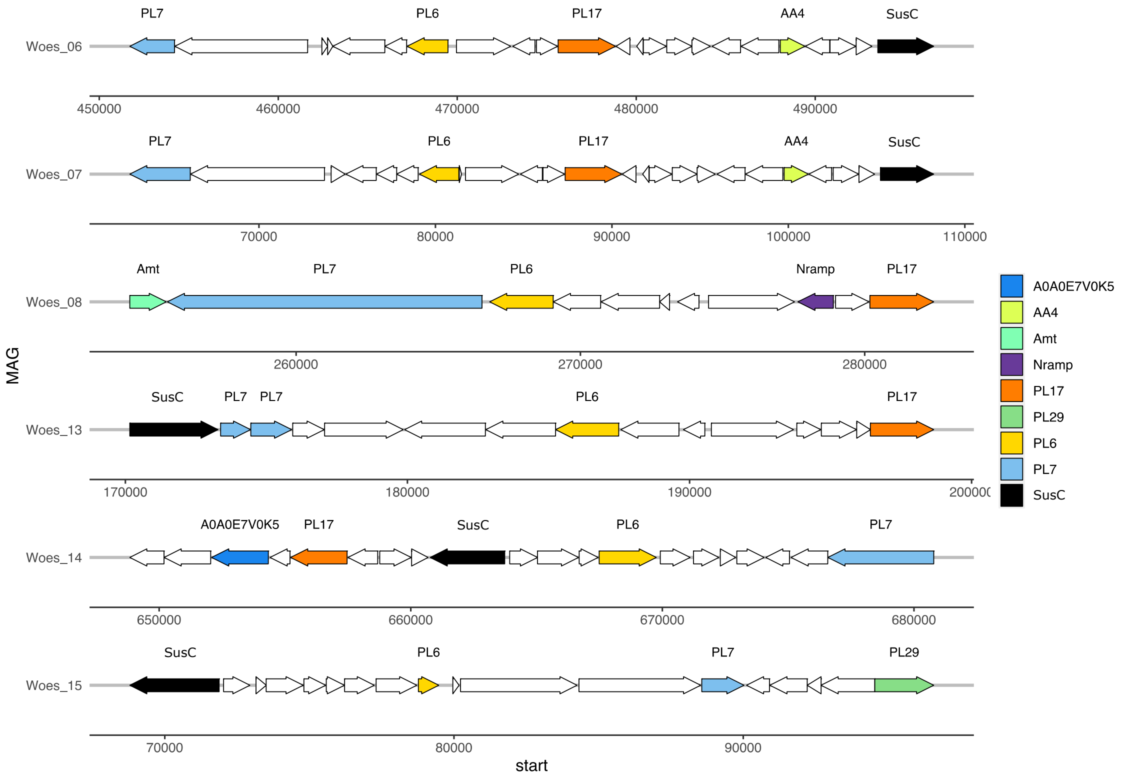

Figure S18: Temporal and geographic distribution of *Woeseiaceae* MAGs in sediment samples. (A) Sediment samples collected in this study. (B) A time-series of Helgoland sediment samples from March to May 2016, including different depth layers (0.5 to 2 cm and 5 to 6 cm). (C) Sediment sample collected from the northern side of the East Frisian Islands in March 2014 in two depth layers (0-2 cm and 2-5 cm). Sandy surface sediment samples from Isfjorden (Svalbard), collected between December 2017 and April 2019 [2], were excluded from the plot as none of the *Woeseiaceae* species identified in this study were detected in those samples.

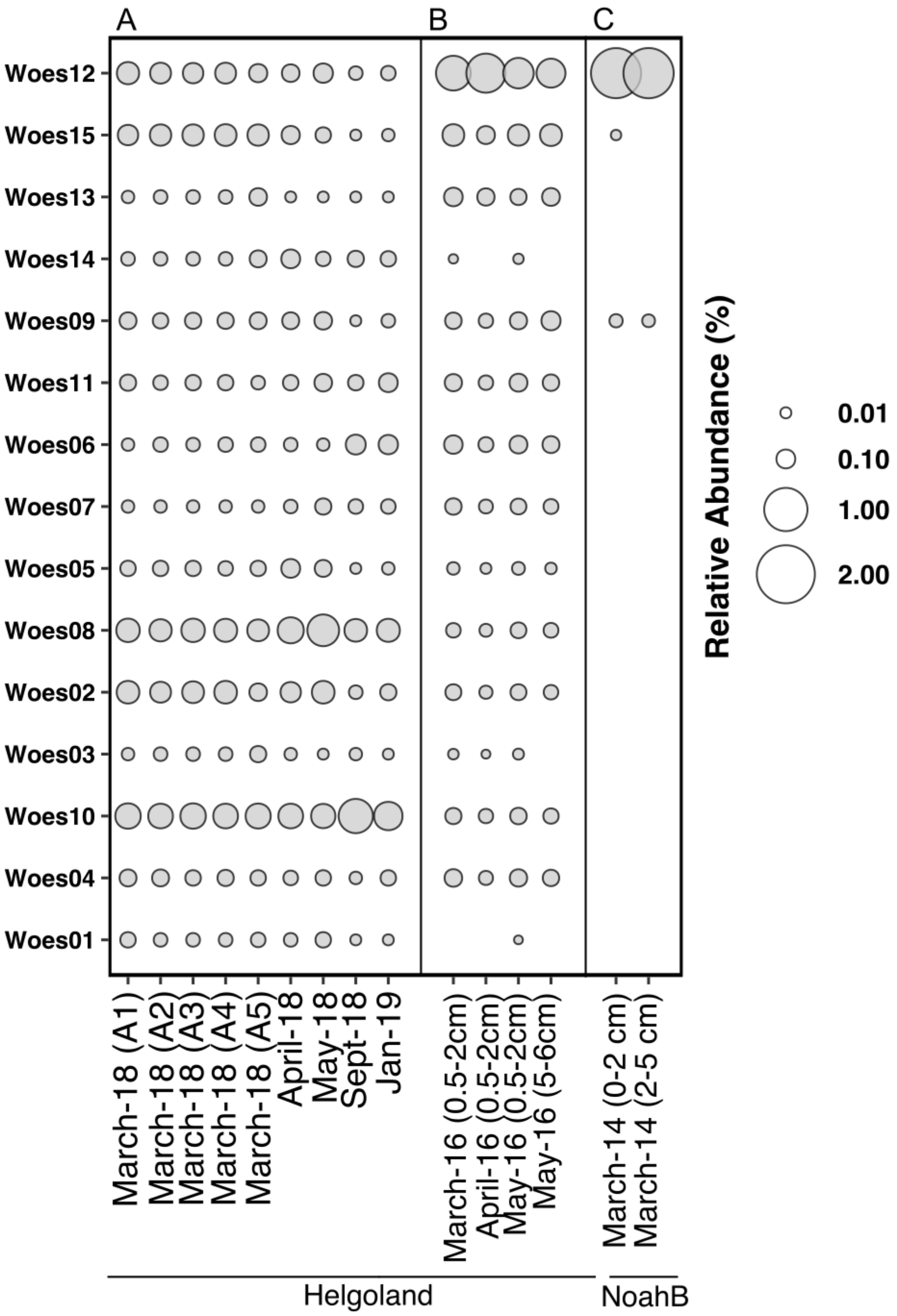

SUPPLEMENTARY REFENCES

[1] Miksch S, Meiners M, Meyerdierks A *et al*. Bacterial communities in temperate and polar coastal sands are seasonally stable. *ISME Comm* 2021;1:29. https://doi.org/10.1038/s43705-021-00028-w

[2] Miksch S, Orellana LH, Oggerin de Orube M *et al*. Taxonomic and functional stability overrules seasonality in polar benthic microbiomes. *ISME J* 2024;18:wrad005. https://doi.org/10.1093/ismejo/wrad005
